# Integrative genomic analyses of European intrahepatic cholangiocarcinoma: Novel *ROS1* fusion gene and PBX1 as prognostic marker

**DOI:** 10.1002/ctm2.1723

**Published:** 2024-06-14

**Authors:** Patrick S. Plum, Timo Hess, Denis Bertrand, Isabelle Morgenstern, Oscar Velazquez Camacho, Christoph Jonas, Christina Alidousty, Britta Wagner, Stephanie Roessler, Thomas Albrecht, Jessica Becker, Vanessa Richartz, Barbara Holz, Sascha Hoppe, Huay Mei Poh, Burton Kuan Hui Chia, Cheryl Xueli Chan, Thushangi Pathiraja, Audrey SM Teo, Jens U. Marquardt, Alexis Khng, Michael Heise, Yao Fei, René Thieme, Sebastian Klein, Jing Han Hong, Simona O Dima, Irinel Popescu, Maria Hoppe‐Lotichius, Reinhard Buettner, Anja Lautem, Gerd Otto, Alexander Quaas, Niranjan Nagarajan, Steve Rozen, Bin Tean Teh, Benjamin Goeppert, Uta Drebber, Hauke Lang, Patrick Tan, Ines Gockel, Johannes Schumacher, Axel M. Hillmer

**Affiliations:** ^1^ Department of General Visceral, Cancer and Transplantation Surgery University of Cologne Faculty of Medicine and University Hospital Cologne Cologne Germany; ^2^ Institute of Pathology University of Cologne Faculty of Medicine and University Hospital Cologne Cologne Germany; ^3^ Department of Visceral Transplant, Thoracic and Vascular Surgery University Hospital of Leipzig Leipzig Germany; ^4^ Center for Human Genetics University Hospital of Marburg Marburg Germany; ^5^ Computational and Systems Biology Agency for Science Technology and Research (A*STAR) Genome Institute of Singapore Singapore Singapore; ^6^ General, Visceral and Transplant Surgery Johannes Gutenberg University Mainz Germany; ^7^ Institute of Pathology University of Heidelberg Heidelberg Germany; ^8^ Liver Cancer Center Heidelberg (LCCH) Heidelberg Germany; ^9^ Institute of Human Genetics University Hospital of Bonn Bonn Germany; ^10^ Cancer Therapeutics and Stratified Oncology Agency for Science Technology and Research (A*STAR) Genome Institute of Singapore Singapore Singapore; ^11^ I Department of Medicine Johannes Gutenberg University Mainz Germany; ^12^ Department of Medicine University Hospital Schleswig‐Holstein Lübeck Germany; ^13^ Department for General Visceral and Transplant Surgery University Hospital Frankfurt Goethe‐University Frankfurt/Main Frankfurt Germany; ^14^ Duke‐NUS Medical School Cancer and Stem Cell Biology Singapore Singapore; ^15^ Division of Medical Science Laboratory of Cancer Epigenome National Cancer Centre Singapore Singapore Singapore; ^16^ Center of Digestive Diseases and Liver Transplantation Fundeni Clinical Institute Bucharest Romania; ^17^ Emeritus of the Division of Transplantation Surgery University Medical Center Mainz Germany; ^18^ RKH Klinikum Ludwigsburg Institute of Pathology and Neuropathology Ludwigsburg Germany; ^19^ Institute of Tissue Medicine and Pathology University of Bern Bern Switzerland; ^20^ Agency for Science Technology and Research (A*STAR) Genome Institute of Singapore Singapore Singapore; ^21^ Center for Molecular Medicine Cologne University of Cologne Cologne Germany

**Keywords:** fusion genes, genomics, intrahepatic cholangiocarcinoma, PBX1, transcriptomics

## Abstract

**Background:**

Cholangiocarcinoma (CCA) is a fatal cancer of the bile duct with a poor prognosis owing to limited therapeutic options. The incidence of intrahepatic CCA (iCCA) is increasing worldwide, and its molecular basis is emerging. Environmental factors may contribute to regional differences in the mutation spectrum of European patients with iCCA, which are underrepresented in systematic genomic and transcriptomic studies of the disease.

**Methods:**

We describe an integrated whole‐exome sequencing and transcriptomic study of 37 iCCAs patients in Germany.

**Results:**

We observed as most frequently mutated genes *ARID1A (14%), IDH1, BAP1, TP53, KRAS*, and *ATM* in 8% of patients. We identified *FGFR2::BICC1* fusions in two tumours, and *FGFR2::KCTD1* and *TMEM106B::ROS1* as novel fusions with potential therapeutic implications in iCCA and confirmed oncogenic properties of *TMEM106B::ROS1 in vitro*. Using a data integration framework, we identified *PBX1* as a novel central regulatory gene in iCCA. We performed extended screening by targeted sequencing of an additional 40 CCAs. In the joint analysis, *IDH1* (13%), *BAP1* (10%), *TP53* (9%), *KRAS* (7%), *ARID1A* (7%), *NF1* (5%), and *ATM* (5%) were the most frequently mutated genes, and we found *PBX1* to show copy gain in 20% of the tumours. According to other studies, amplifications of *PBX1* tend to occur in European iCCAs in contrast to liver fluke‐associated Asian iCCAs.

**Conclusions:**

By analyzing an additional European cohort of iCCA patients, we found that PBX1 protein expression was a marker of poor prognosis. Overall, our findings provide insight into key molecular alterations in iCCA, reveal new targetable fusion genes, and suggest that *PBX1* is a novel modulator of this disease.

## Graphical abstract

Genomics of intrahepatic cholangiocarcinoma in European patients is understudied. *FGFR2::KCTD1* and *TMEM106B::ROS1* are novel fusions. Data integration reveals *PBX1* as a central gene for intrahepatic cholangiocarcinoma. *PBX1* expression correlates with short overall survival.

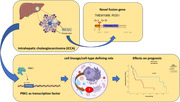

## 1 INTRODUCTION

Intrahepatic cholangiocarcinoma (intrahepatic CCA or iCCA), a cancer of the bile ducts, is the second most common primary hepatic cancer, accounting for 10−20% of liver cancers.^1^, ^2^ iCCA has a poor prognosis, with a 5‐year survival rate of less than 10%.^3^, ^4^, ^5^ Between 1979 and 2000, the age‐standardized incidence rate of CCA increased tenfold in the United Kingdom,^6^ and mortality due to CCA continues to rise globally.^7^ Patients diagnosed at an early, locally limited stage of the disease are eligible for surgery, the only curative treatment option.^8^ However, most patients are diagnosed at an advanced stage, without adequate options for standard care. Therefore, only approximately 25% of all tumours are resectable at the time of diagnosis.^9^, ^10^ Even with successful resection and an appropriate R0, intrahepatic recurrence of CCA occurs within the first two to three years postoperatively in 49% to 64% of patients.^11^ Adjuvant chemotherapeutic approaches have been designed to stabilize the tumour‐free status, but the results from the majority of phase III trials have been disappointing, showing no or no significant survival benefit for patients who receive additional chemotherapeutic regimens.^12^, ^13^, ^14^, ^15^ Therefore, palliative treatment is administered to most patients after diagnosis.^16^, ^17^, ^18^

Several risk factors for iCCA have been identified, including parasitic infections, primary sclerosing cholangitis, biliary duct cysts, hepatolithiasis, and toxins.^19^ In particular, the liver fluke (*Opisthorchis viverrini*), a parasite that infects the bile duct, has been identified as a risk factor for CCA and is responsible for substantial regional differences in CCA incidence rates.^20^, ^21^ This is because *O. viverrini* is prevalent in Southeast Asia where a local habit of eating raw freshwater fish prevails. The fish is the intermediate host of *O. viverrini*.^22^

Over the last decade, there has been significant progress in our understanding of the molecular characteristics of iCCA. The majority of iCCA samples analyzed by whole exome/genome sequencing were derived from Asian patients, including three large studies with 103, 137, and 173 patients.^23^, ^24^, ^25^ Nonsilent mutations in *TP53* have been found at high frequencies in Asian populations ranging from 10% to 42%.^23^, ^24^, ^25^, ^26^ Fewer iCCAs of patients of European descent have been exome/genome sequenced, with 8 to 32 patients per study (Jiao et al.^27^ [*n* = 32]; Sia et al.^28^ [*n* = 8], Jusakul et al.^25^ [*n* = 30]; Farshidfar et al.^29^ [*n* = 27]), where *TP53* mutation frequencies ranged from 6% to 11%.^26^, ^27^, ^29^ *ARID1A, KRAS, BAP1, IDH1*, and *SMAD4* showed nonsilent mutation frequencies >10% in some cohorts, and other cancer driver genes, including *ATM, PIK3CA*, and *NRAS*, have been described to have lower mutation frequencies for iCCA.^23^, ^24^, ^26^, ^27^, ^29^ Mutations in *TP53* and *KRAS* and deletions in *CDKN2A* are predictors of short overall survival.^30^ Mutations in the metabolic enzymes *IDH1* and *IDH2* which are enriched in tumours with a high expression of mitochondrial genes,^24^, ^27^, ^29^, ^31^, ^32^ are potentially therapeutic targets.^33^ In addition, *FGFR2* fusion genes with frequencies of up to 45% in iCCA,^28^, ^29^, ^34^, ^35^ constitute new targets for therapy^36^ and can be regarded as a breakthrough for iCCA patient management.

Although *IDH1/2* and *FGFR2* alterations provide new options for targeted treatment,^37^, ^38^ this is not the case for a large proportion of iCCAs patients. There is a need for a better understanding of the molecular processes leading to iCCA, particularly in European patients who are understudied at an exome‐wide level, to develop treatment strategies that might target not only driver genes but also cell lineage‐specific pillars of iCCA. Therefore, we performed whole‐exome sequencing and transcriptomic analysis of 37 German patients with iCCA and carried out an integrative network‐based analysis to identify new central nodes of iCCA. We identified previously described and new oncogenic fusion genes for iCCA, including *FGFR2::KCTD1* and *TMEM106B::ROS1*. Subsequently, we screened an additional collection of CCAs by targeted sequencing and identified the transcription factor *PBX1*, as a central gene with recurrent genomic alterations. Investigating an additional German cohort for PBX1 protein expression in CCA revealed an association between PBX1 expression and shorter overall survival. Overall, to the best of our knowledge, this is the largest exome‐sequenced iCCA cohort of European descent with new fusion genes for iCCA and PBX1 as prognostic factors.

## 2 MATERIALS AND METHODS

### 2.1 German iCCA patients (discovery screen)

Forty patients (20 females and 20 males) diagnosed with iCCA were recruited as the discovery cohort. The median age of the patients on the date of the first surgery was 65.5 years (32−84 years). All patients underwent curative surgery at Mainz University Medical Center (Germany) and provided informed consent. Freshly frozen tumours and matched normal tissues comprising normal liver tissue or whole blood were obtained during surgery and used for the experiments. In one patient (CCC‐26), the tumour material of a relapse (CCC‐26a), in addition to the tissue of the primary tumour, was obtained. Whole exome sequencing (WES) was successfully performed on 37 tumour/normal pairs and CCC‐26a, and 35 tumours, and CCC‐26a were analyzed by single nucleotide polymorphism (SNP) array. Transcriptome analysis was performed on 31 tumours and CCC‐26, resulting in high‐quality data for 22 tumours plus CCC‐26a and 9 normal liver tissues, and 25 tumours were screened for fusion genes by Archer (Table S1). All clinical and pathological characteristics of the initial discovery screen and the overview of the genetic methods applied are summarized in Table 1.

**Context and significance**

Intrahepatic cholangiocarcinoma is a cancer of the bile duct with a poor prognosis due to limited treatment options. Most genomic studies on bile duct cancer have been performed in Asian populations so our understanding of common mutations in European patients is lagging behind. In the present study, bile duct tumours of German patients have been analyzed for mutations and gene activities. Chromosome breaks resulting in new gene fusions that likely drive cancer development have been identified in two tumours. They might provide targets for treatment. Further, PBX1, a transcription factor that can turn on other genes has been found to be active in some tumours. Patients with PBX1 activity have a poor prognosis. PBX1 might be a new biomarker.

**TABLE 1:** Characteristics of the initial German iCCA patients from the discovery screen.

	Total number of patients (*n* = 40)
**Clinical characteristics**	
**Sex**	
Male	20
Female	20
**Age**	
Median	65.5 years
(minimum—maximum)	(32–84 years)
**Postsurgical survival**	
Median	413 days
(minimum—maximum)	11–4003 days
**Comorbidities**	
Diabetes mellitus	
Yes	7
No	33
Obesity	
Yes	2
No	38
Hypertension	
Yes	12
No	28
Liver diseases	
None	33
Condition after cholecystectomy	5
Cholelithiasis	1
Primary sclerosing cholangitis	1
**Histopathological characteristics**	
**pT‐category**	
pT1	14
pT2	11
pT3	7
pT4	1
n/a	7
**pN‐category**	
pN0	25
pN1	4
n/a	11
**M‐category**	
M0	24
M1	1
n/a	15
**Grading**	
G1	1
G2	24
G3	2
G1‐2	1
G2‐3	2
n/a	10
**R‐status**	
R0	25
R1	3
n/a	12
**Genetic analyses**	
**WES library preparation**	
**Tumour/normal (blood)**	
Successful	37
Unsuccessful	3
**SNP‐Array**	
**Tumour**	
Successful	35
Unsuccessful	5
**Transcriptomics**	
**Tumour**	
Successful	22
Unsuccessful	18
**Normal liver**	
Successful	9
Not attempted	31
**Fusion gene analysis**	
**Tumour**	
Successful	25
Unsuccessful	15

Abbreviations: n/a, not available; WES, whole exome sequencing; SNP, single nucleotide polymorphism.

### 2.2 Discovery screen

A detailed description of the sample preparation, WES, single nucleotide variants (SNVs)/indel validation by Sanger and Illumina sequencing, identification of recurrently mutated genes, and SNP array‐based copy number analysis can be found in the Supporting information Methods.

### 2.3 Fusion gene analysis using multiplex single primer extension‐based RNA‐sequencing

For the 25 tumours, sufficient RNA was available for fusion gene‐directed sequencing using the FusionPlex Kit for Illumina (Archer) and FusionPlex Lung Panel (Archer) according to the manufacturer's recommendations. RNA (200 ng) was used as the input. Libraries were sequenced at 2 × 150 bp using the Illumina NextSeq 550 platform. The data were analyzed using Archer Suite Analysis v5.0.4, and v5.1.3, for additional sensitivity (Table S11, Figures S1–S3).

### 2.4 Expression array

For transcriptome‐wide expression analysis, 200 ng of RNA isolated from tumour and normal liver tissues was subjected to transcriptome‐wide expression analysis using the HumanHT‐12 v4 Bead Array (Illumina) according to the protocols provided by the manufacturer. Of the 32 tumour samples and 10 paired normal samples, sufficient RNA with an RNA integrity value >6 was available for array analysis, including one relapse pair. The intensity values for each transcript were obtained using the Gene Expression Module (v. 1.9.0) of GenomeStudio (Illumina). Low‐quality array data for the nine tumours were excluded from further analysis. The mean intensity values of the remaining samples were used to assign the fold change (FC) between the tumour and normal sample sets. These data were used as inputs for OncoIMPACT. For differential expression analysis, the relapse tumour normal pair (CCC‐026a) was excluded, resulting in 22 tumour and nine control samples. Differential expression between tumour and normal samples was determined using the limma Bioconductor package,^39^ and the corresponding p values were corrected for multiple testing using the Benjamini–Hochberg false discovery rate (FDR) method. An adjusted *P*‐value threshold of 0.05 and a log2‐fold change ≥2 were used to determine differential gene expression (Table S12).

### 2.5 Gene set enrichment analysis

Gene set enrichment analysis (GSEA) was performed on a preranked list of differentially expressed genes when comparing CCA with normal transcriptomic data. GSEA was performed using GSEA v4.0.1 software.^40^, ^41^ The MsigDB gene sets “Hallmark” and “C6‐Oncogenic” were used to identify genes enriched in these pathways. All gene set files for this analysis were obtained from the GSEA website (www.broadinstitute.org/gsea/). An enrichment map was used to visualize the GSEA results. The enrichment score (ES) and false discovery rate (FDR) values were applied to sort the enriched pathways after 1000 gene set permutations were performed for the analysis (Table S13).

### 2.6 OncoIMPACT

We used OncoIMPACT version 0.9 with default parameters. All the samples for which SNV/indel and CNA data were available were included in the analysis (sample CCC‐026a was excluded). Differential expression was computed as described in the Expression Array section. All somatic indels and SNVs annotated as missense, nonsense, or splice sites were included in the point mutation matrix. For all genes in each sample, we computed the difference between the estimated gene copy number and the estimated sample ploidy: genes with a value less than −1 were considered to be deleted, genes with a value higher than 3 were considered to be amplified, and all other genes were considered to have neutral copy numbers. Genes from the X and Y chromosomes were excluded because of the difficulty in estimating their copy number. We reported the driver gene list inferred using the OncoIMPACT stringent mode (Table S14).

### 2.7 Extended screening by deep amplicon sequencing

We analyzed 49 paired tumour/normal genomic DNA samples (nine pairs overlapping with the discovery set were used to evaluate the quality of SNV and CNA calling, and 40 unrelated pairs for the extended screen; Table S15). Amplicons were generated using the GeneRead DNAseq Panel PCR Kit V2 (QIAGEN) and GeneRead Custom Panel CNGHS‐00906X‐2135 (QIAGEN), comprising targets across 44 genes (Table S16), according to the manufacturer's protocol. The amplicons generated for each sample were pooled and purified using AMPure XP magnetic beads (Agencourt Bioscience Corporation) and subjected to an automated protocol on the QIAcube (QIAGEN) comprising end repair, addition of an A‐tail, ligation of a custom adaptor using the GeneRead DNA Library I Core Kit (QIAGEN), and size selection using the GeneRead Size Selection Kit (QIAGEN). PCR was then performed on the size‐selected adaptor‐ligated DNA using Phusion High‐Fidelity PCR Master Mix (Thermo Fisher Scientific) together with a universal primer (5‐’ AATGATACGGCGACCACCGAGATCTACACTCTTTCCCTACACGACGCTCTTCCGATC*T3) and a primer with an 8‐bp index (5′CAAGCAGAAGACGGCATACGAGAT‐Index‐GTGTGACTGGAGTTCAGACGTGTGCTCTTCCGATC*T3'), resulting in each sample having a unique barcode. The following program was used: (1) initial denaturation at 98°C for 40 s, (2) 10 cycles of 12 s at 98°C, 30 s at 65°C, and 30 s at 72°C, and (3) 72°C for 5 min. The individual barcoded libraries were purified using the QIAquick PCR Purification Kit on the QIAcube (QIAGEN), analyzed using the DNA 1000 Assay on the Bioanalyzer or TapeStation (Agilent Technologies), quantified by qPCR with the KAPA Library Quantification Kit for Illumina platform (KAPA Biosystems) on a LightCycler 480 (Roche), and pooled at 5 nM per sample into two multiplex libraries comprising 49 tumour and 49 normal samples. Each library was sequenced in one lane of a HiSeq Rapid 2 × 151 on an Illumina HiSeq2000 sequencer. The NGS data were processed using the same pipeline as used for WES but without the removal of PCR duplicates (Table S17). For calling SCNAs, Quandico v1.13 was used with standard settings^42^ (Table S18). Nine tumour/normal pairs from the German iCCA cohort were used to validate the copy number calling, as this approach is less robust for amplicon‐based targeted sequencing (Figure S4).

### 2.8 In vitro analyses of the effects of PBX1 on nonmalignant and malignant human biliary cell lines

To evaluate a specific PBX1‐dependent cancer‐relevant phenotype, we utilized MMNK‐1, a nonmalignant, immortalized biliary cell line^43^ as well as HuH‐28^44^ and HuCCT‐1,^45^ which are malignant cholangiolar tumour cell lines for further in vitro experiments (for a detailed explanation see the Supporting information Notes/Methods).

Using quantitative reverse transcription PCR (qRT‐PCR), the expression of different *PBX1* splicing variants was characterized. Knockdown cell lines were generated by treatment with short hairpin RNAs, and predominant *PBX1_202* overexpressing cells were generated via stable transduction. The effect on PBX1 expression was evaluated via qRT‐PCR and Western blotting. These cell lines were then tested by cancer assays for proliferation, invasion, colony formation, and chemotherapy resistance. Furthermore, all cell lines were subjected to RNA sequencing.

### 2.9 Tissue microarray immunohistochemistry from an independent patient cohort with iCCA

To validate our findings, we performed immunohistochemical analyses of a well‐characterized CCA cohort of European descent via tissue microarray (TMA).^46^ The following exclusion criteria were used: (1) administration of systemic therapy prior to surgery to avoid bias in the survival analysis. (2) Survival <14 days after surgery to exclude short‐term deaths due to surgical complications. A total of 36 patients with iCCA fulfilled the inclusion criteria. Four‐micrometer sections of the TMA blocks were transferred to an adhesive‐coated slide (Instrumedics Inc.) for staining. Immunohistochemistry (IHC) was performed on TMA slides using a primary antibody against PBX1 (clone: HPA003505, Sigma‐Aldrich Inc., dilution: 1:500, citrate buffer) with a Bond Max automated system (Leica). This marker showed a nuclear staining pattern in the TMA. Two pathologists (U.D. and B.J.W.) manually performed the IHC analysis. Staining was assessed using a two‐tier scoring system (0 or 1). A score of 0 indicated the absence of PBX1‐staining while PBX1 protein was detected in samples with a score of 1. The human endometrium served as a positive control.

SPSS v26.0 (IBM) was used for statistical analysis and graphical presentation of the results. The interdependencies between staining and clinical data were calculated using the chi‐squared test and Fisher's exact test. Survival curves were plotted using the Kaplan–Meier method and analyzed using the log‐rank test. All tests were two‐tailed. Statistical significance was set at *P* < 0.05.

### 2.10 Statistical analysis

GraphPad Prism software version 9 was used for statistical analysis. Data from cell line experiments (Supporting information Data) are presented as the mean ± standard deviation (SD), and statistical significance was evaluated using a two‐tailed Student's *t*‐test. ANOVA (analysis of variance) was used to measure significant differences between multiple groups, and *P*‐values < 0.05 were considered to indicate statistical significance.

For survival analysis, Kaplan–Meier survival plots were generated for overall survival (OS) and compared using the log‐rank test. OS was defined as the time from tumour resection to death. The threshold for statistical significance was predefined as two‐sided *P *< 0.05.

### 2.11 Ethical approval

All procedures were in accordance with the ethical standards of the responsible committee on human experimentation (institutional and national) and the Helsinki Declaration of 1964 and later versions. Informed consent was obtained from all patients. This study was approved by the ethics committee of the State Medical Association Rhineland‐Palatinate (837.326.08(6323)).

## 3 RESULTS

### 3.1 Genomic landscape of European iCCA

**FIGURE 1:**
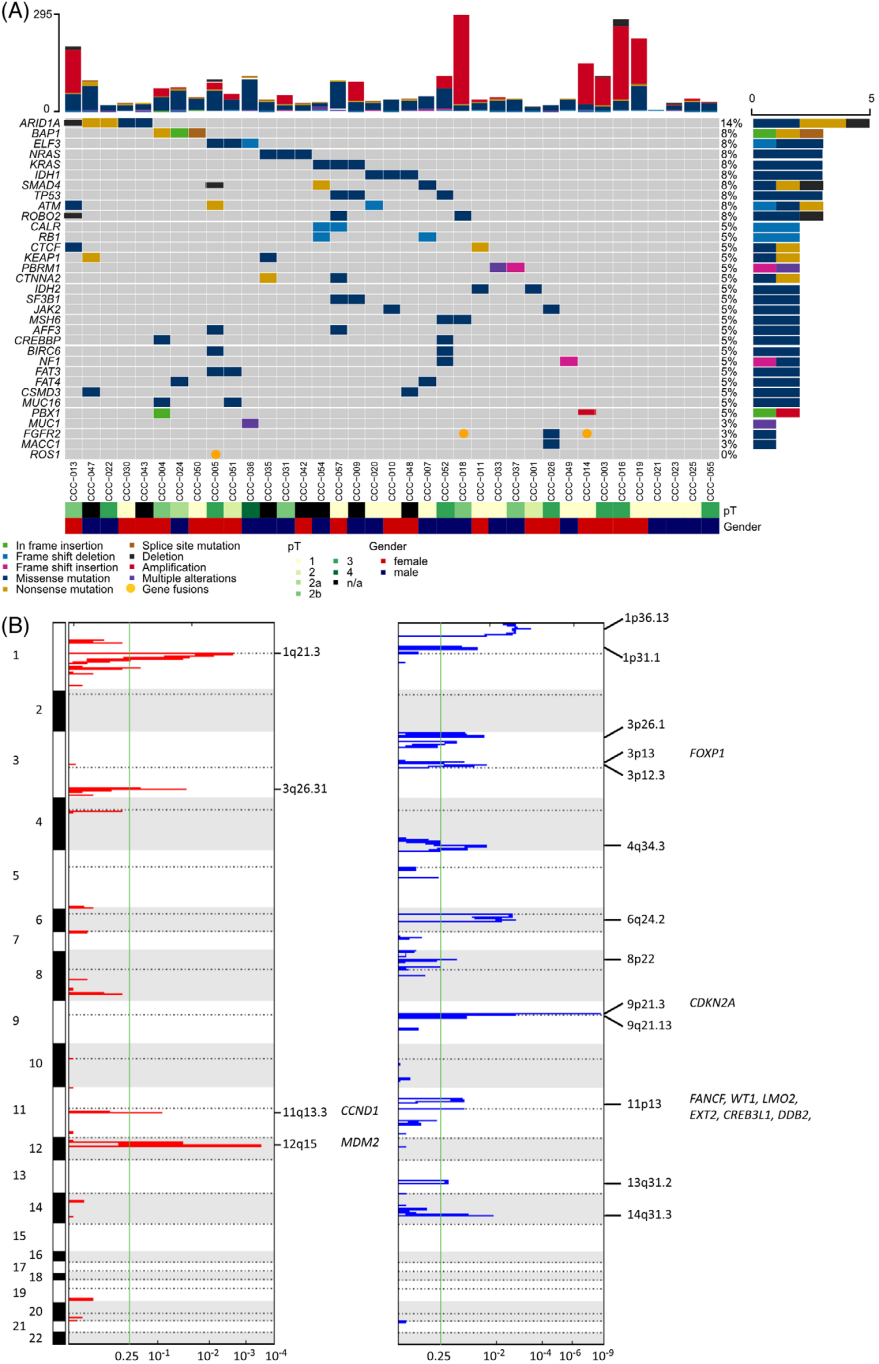
Genomic characterization of 37 intrahepatic CCA samples from Germany. (A) Oncoplot based on whole exome sequencing showing the most frequently altered genes (rows) for 37 iCCAs (columns). Displayed genes were filtered based on MutSigCV analysis and cancer gene census (Methods, Table S6), and SCNA information was subsequently added. The numbers of somatic SNVs and indels as well as gene‐based amplifications and deletions per tumour are indicated at the top, tumour stage and sex are indicated at the bottom, and the numbers of somatic alterations per gene are illustrated on the right. (B) GISTIC plot showing the recurrently observed copy number gains (red) and losses (blue) across the genome based on SNP array analysis (top to bottom, chromosomes indicated on the left). The *q*‐values indicating significant recurrence are provided at the bottom. The cytobands of the significant results crossing the green lines are depicted next to the peaks (*q* < 0.25). The Cancer Gene Census genes within the narrow peak regions are indicated in italics on the right. pT, pathological tumour stage.

We sequenced the exomes of 37 tumour/normal pairs of German patients with iCCA (Supplementary Tables S1 and S2) and identified 2749 somatic SNVs and short insertions/deletions (indels). These included 1262 missense SNVs (45.9%), 492 synonymous SNVs (17.9%), 119 indels (4.3%), 90 nonsense SNVs (3.3%), 28 splice sites (1%), and 758 SNVs located within exon adjacent intronic regions (27.6%; Tables S3–S5). Overall, we detected an average of 39 nonsilent mutations per sample and .94 exonic mutations per megabase (Mb). This finding is consistent with the mutation rates reported for liver fluke‐negative CCA (1.39 SNVs/Mb [including silent]^25^;), but lower than those reported for liver fluke‐negative CCA and gallbladder cancers from Japan (2.6 somatic SNVs/Mb^24^;) and another study in which 2.6 mutations/Mb were estimated by targeted sequencing of iCCAs.^30^ The difference in the mutation burden might be due to differences in aetiology and analytical procedures. We ranked genes for their relevance in iCCA based on small mutations using MutSigCV and considered their cancer relevance (Figure 1A, Methods, Table S6).

Among the nominally significantly mutated genes, *ARID1A* was most frequently mutated, with four nonsilent small mutations and one deletion (five tumours, 14%). *KRAS* or *NRAS* were mutated in six tumours (16%), and *IDH1* or *IDH2* were mutated in 5 tumours (14%). Several genes, including *BAP1*, *ELF3, TP53, SMAD4, ATM and ROBO2*, exhibited genomic alterations in three tumours (8%) (Figure 1A). The mutation frequencies for *IDH1, BAP1, ARID1A* and *PBRM1* were lower than those reported in recent analyses of iCCA using targeted sequencing^30^, ^47^, ^48^ which might be due to the inclusion of unresectable cases and deeper sequencing in these studies.

Furthermore, we analyzed tumours using SNP arrays for somatic copy number alterations (SCNAs, Tables S7–S9). More than half of the tumours tended to accumulate focal amplifications that did not affect the overall diploid state (52.7% of tumour samples). In total, ten tumours were tri‐ and six were tetraploid. We identified four recurrent copy gains and 13 deleted genomic regions (peaks) with a significant FDR (*FOXP1, CDKN2A, FANCF, WT1, LMO2, EXT2, CREB3L1, DDB2)* (*q* < 0.25; Figure 1B, Table S10). The copy gain regions were located on 1q21, 3q26, 11q13, and 12q15, with 33 genes located in narrow peak regions, including the Cancer Gene Census listed genes *CCND1 and MDM2*, both of which are frequently amplified in an Asian CCA cohort.^24^ The gain of *MDM2*, a negative regulator of *TP53*, emphasizes the role of the gatekeeper *TP53* in iCCA transformation. We did not find chromosomal arms 2p and 2q among the frequently amplified regions as reported by Jukasul et al.^24^

Narrow peaks of the deleted regions included 572 genes at 1p36, 1p31, 3p26, 3p13, 3p12, 4q34, 6q24, 8p22, 9p21, 9q21, 11p13, 13q31, and 14q31. These narrow peaks included *CDKN2A, CREB3L1, DDB2, EXT2, FANCF, FOXP1, LMO2*, and *WT1* as cancer census genes. Wider regions included *ARID1A* and *BAP1*. By analyzing the copy number data derived from two other studies on iCCA^26^, ^49^ several significantly amplified and deleted genomic regions were validated. *CDKN2A* is the gene most frequently affected by SCNAs and is deleted in five tumours (14%), a gene in which deletions predict poor outcomes.^30^

When stratifying patients according to clinical features, we observed a particularly high mutation burden of 181 small somatic variants (SNVs/indels; average among 37 iCCA patients was 74), including two missense mutations in *MUC1* and frameshift mutations in *ELF3* and *AURKAIP1*, in‐patient CCC‐036 with primary sclerosing cholangitis (Table S1). This tumour did not harbor any genes affected by SCNAs. After cholecystectomy, six patients had an average of 105 small somatic variants including two nonsilent mutations in *BAP1*, two in *MUC1* (patient CCC‐036) and one each in *ELF3, FAT4, KRAS, MSH6, MUC16, PBX1, RB1*, and *SMAD4*. Five patients had liver diseases (polycystic liver disease, liver fibrosis, liver cirrhosis, or chronic hepatitis B) with an average of 97 small somatic variants, including nonsilent variants in *ARID1A, CSMD3*, and *KEAP1* and one copy gain of *PBX1*. Seven patients had diabetes mellitus type 2, with an average of 97 somatic small variants, including nonsilent variants in *BAP1, ELF3, FAT4, KRAS*, and *TP53*. With the exception of sclerosing cholangitis, the comorbidities did not show obvious associations with particular genomic alterations.

Overall, at least one cancer driver gene^50^ (CGC downloaded 15 September 2022) was affected by somatic mutations or copy number gains or losses in 36 of the 37 samples, with the low‐tumour‐content sample CCC‐021 being the only one without a detected driver alteration.

### 3.2 Identification of *FGFR2* fusion genes and a new *ROS1* fusion gene for iCCA

**FIGURE 2:**
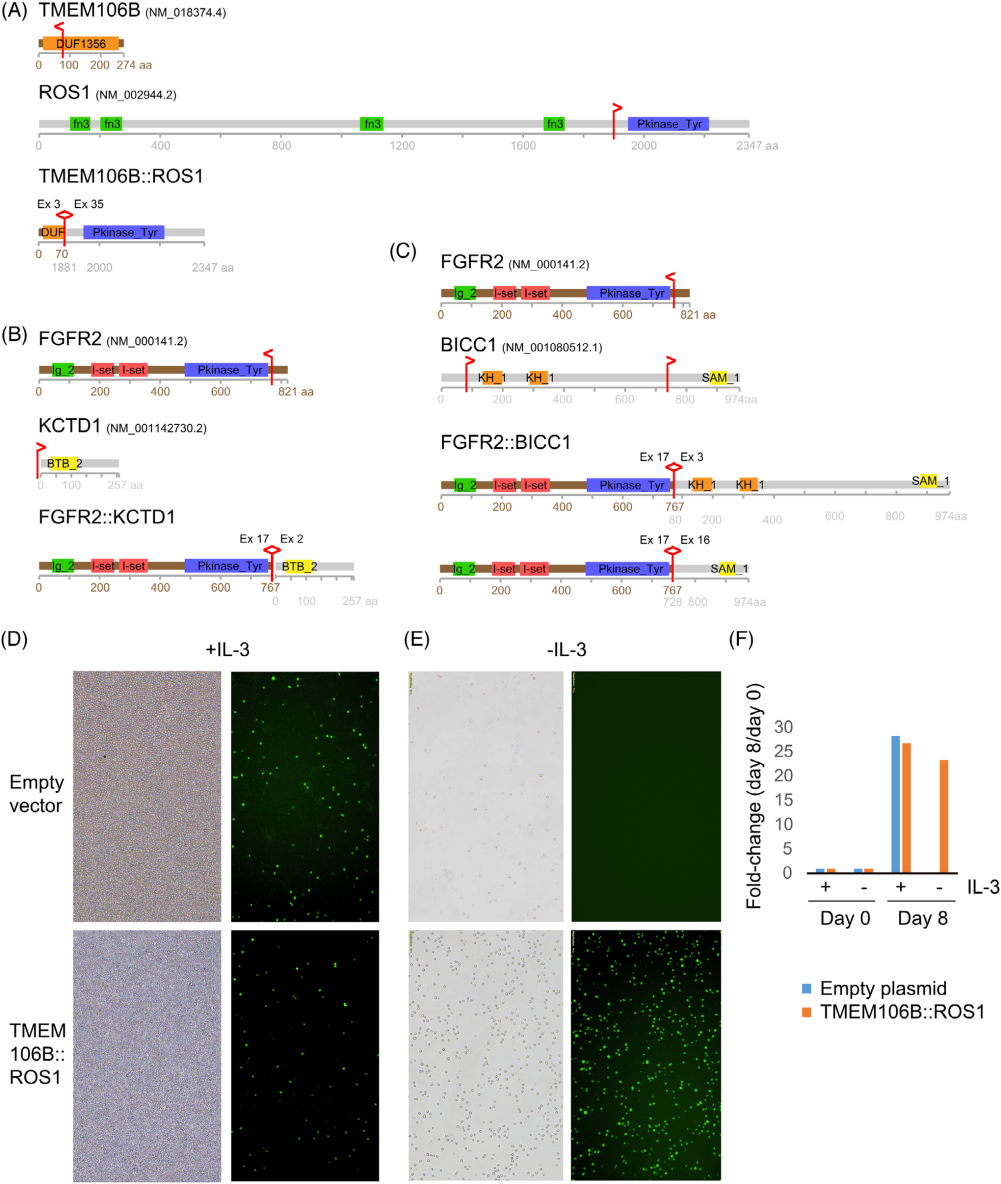
Fusion genes identified in iCCA from Germany. (A–C) Schematic representation of the predicted fusion proteins based on cDNA sequencing. The vertical red lines indicate break points, with the arrowhead pointing to the direction of the part of the protein that is fused to the other protein. DUF1356, domain of unknown function; fn3, fibronectin type III domain; Pkinase_Ty, protein tyrosine kinase; Ig_2, immunoglobulin domain; I‐set, immunoglobulin I‐set domain; BTB_2, BTB/POZ domain; KH_1, KH domain; SAM_1, sterile alpha motif domain; protein domain information derived from www.cbioportal.org. (D–F) Ba/F3 cells were stably transduced with the *TMEM106B::ROS1*‐fusion encoding MIGR1 plasmid or the empty MIGR1 plasmid as a control. Images were taken at 100× magnification. Left: bright field images; right: fluorescence images. (D) Pictures were taken four days after transduction in media supplemented with IL‐3. (E) Images were taken eight days after IL‐3 withdrawal. (F) Quantification of Ba/F3 cells at day 8 compared with day 0.

Since fusion genes have been established in recent years as an important category of driver genes for CCA constituting targets for therapy,^51^, ^52^, ^53^ we screened tumours with sufficient RNA (*n* = 25) for fusion genes using a multiplex single‐primer extension‐based RNA‐sequencing approach (Methods). We identified three tumours with *FGFR2* fusions, two of which had *FGFR2* fusions in combination with *BICC1*. In the first case, *FGFR2* exon 17 was fused to *BICC1* exon 3 as previously reported^34^, ^54^, ^55^, ^56^ for which oncogenic activity was assumed. In the second case, *FGFR2* was fused to exon 16 of *BICC1*, a rare configuration found in data from The Cancer Genome Atlas (Firehose Legacy data, derived from FusionGDB2 [https://compbio.uth.edu/FusionGDB2/]). Furthermore, we observed an *FGFR2*[ex17]::*KCTD1*[ex2] fusion with reported evidence of pro‐proliferative activity.^57^, ^58^ Interestingly, we identified a fusion between *TMEM106B[ex3]::ROS1[ex35]*, which has been reported in non–small cell lung cancer^59^ but not in CCA (Figure 2, Table S11, Figures S1–S3). The exons involved in the fusions have been described as fusion sites for lung cancer^59^, ^60^, ^61^ resulting in the inclusion of the tyrosine kinase domain of *ROS1* and *FGFR2*, suggesting oncogenic functions. The predicted FGFR2 fusion proteins contain the BTB domain of KCTD1 and the SAM domain of BICC1, which mediate protein‐protein interactions, resulting in FGFR2 dimerization, autophosphorylation, and activation.^61^ For *ROS1* fusions, the mechanism of action is less clear, but conformational changes are thought to activate *ROS1*, where the loss of all or most fibronectin domains is thought to be responsible for acquiring the activated state.^60^ *ROS1* rearrangements are considered to be very rare events in iCCA (1.1%).^62^ Overall, 16% of the tested tumours (4/25) harboured oncogenic fusion genes and we identified *TMEM106B::ROS1* as a new fusion gene for CCA.

To investigate the potential oncogenic role of the *TMEM106B::ROS1* fusion protein, we stably introduced it into the interleukin 3 (IL‐3)‐dependent Ba/F3 murine hematopoietic cell line together with green fluorescent protein (GFP; Figure 2D–F). Due to their IL‐3 dependency, Ba/F3 cells die shortly after withdrawal of exogenous IL‐3 unless the ectopic expression of an oncogenic driver renders their survival and proliferation independent of IL‐3. As shown in Figure 2E, eight days after the withdrawal of IL‐3, the number of Ba/F3 cells transduced with the control vector drastically decreased (top), despite the seeding of equal numbers of cells on day one of withdrawal. Furthermore, no fluorescence could be detected in the remaining cells. On day eleven of IL‐3 withdrawal, no intact cells were observed, but only cell debris was present (data not shown). In contrast, *TMEM106B::ROS1*‐expressing Ba/F3 cells continued to proliferate (Figure 2D, E, bottom) and strikingly, all surviving cells were GFP‐positive, indicating that only the transduced cells survived in the absence of IL‐3, supporting the oncogenic characteristics of the fusion protein.

### 3.3 Transcriptomic characteristics of European iCCA

**FIGURE 3:**
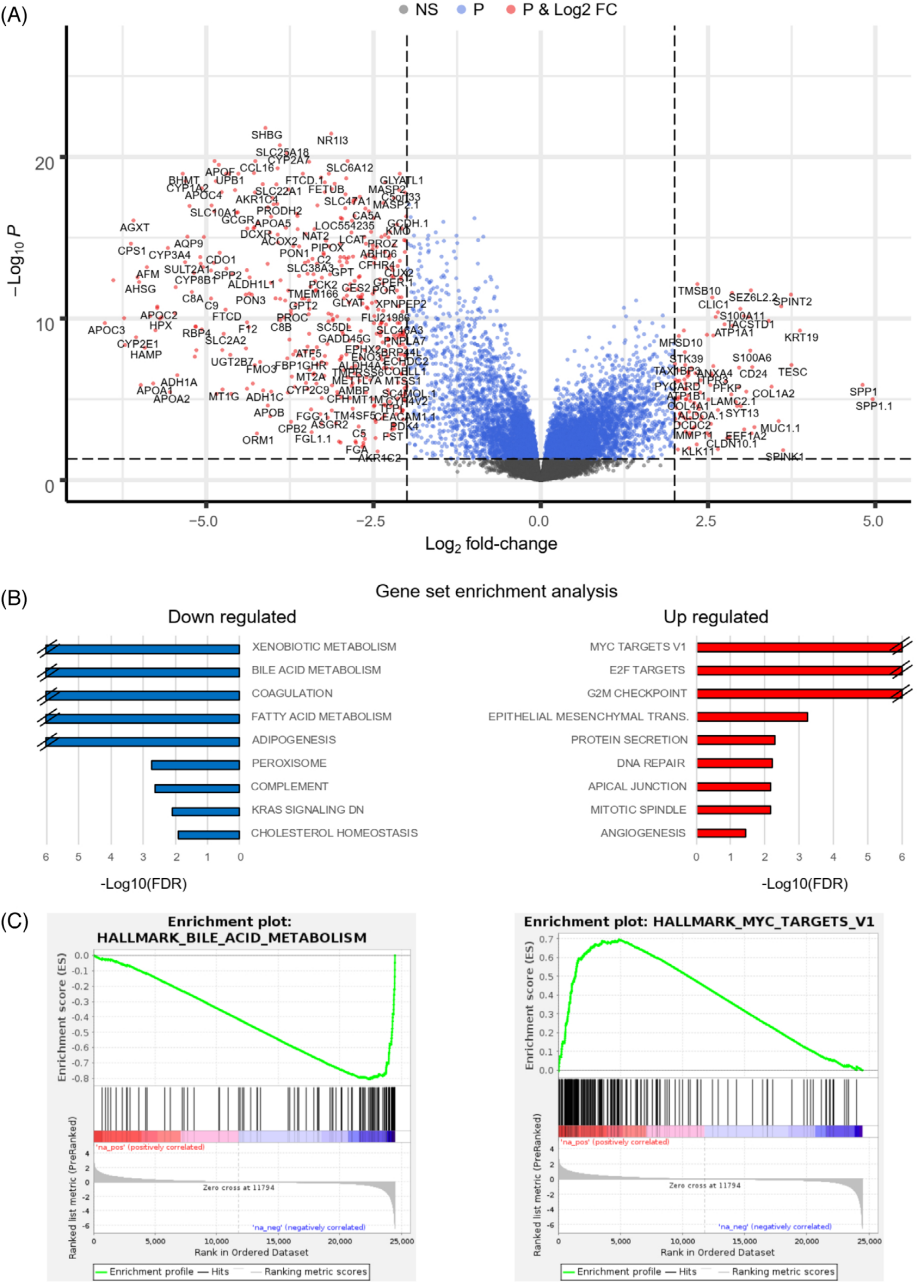
Expression analysis of iCCA from Germany. The transcriptomes of 22 CCA patients were compared with those of nine normal liver controls by expression array analysis. (A) Volcano plot indicating genes that are differentially expressed between tumour and normal tissues are indicated as dots with log2‐fold changes on the *x*‐axis and −log10 *P*‐value on the *y*‐axis. An adjusted *P*‐value (*P*) threshold of 0.05 and a log2‐fold change (log2 FC) ≥2 (dashed lines) were used to determine differential gene expression (red dots). The names of a randomly selected subset of significant DEGs are indicated, to avoid overlapping fonts. (B) Preranked gene set enrichment analysis of differentially expressed genes is indicated for downregulated and upregulated genes, respectively. Gene sets with an FDR *q*‐value < 0.05 are shown as −log10 (FDR) on the *x*‐axis. The values are capped at 6. For a complete list, see Table S13. (C) Gene set enrichment profile of selected downregulated (left) and upregulated (right) gene sets. The vertical bars (middle) indicate the positions of genes in the set within the ranked distribution of differential expression values (bottom), resulting in enrichment scores (top).

As a basis for integrative analysis, we analyzed tumour samples from 22 patients and 9 normal tissues using a transcriptomic array (Methods) and defined the iCCA profile by differential expression analysis between tumour and normal tissues. Among the top upregulated genes in iCCA were typical epithelial marker genes, including *KRT19, MUC1, CLDN10*, and *EPCAM* (*TACSTD1*), and extracellular matrix genes, including *COL1A1, COL1A2*, and *MMP7*, reflecting the nature of epithelial cancer (Figure 3A, Table S12). *SPP1* (log2 fold change [FC] = 5) was the most highly upregulated gene, followed by *KRT19* (log2FC = 4.8).

To classify iCCA transcriptomic dysregulation in the context of cancer hallmarks, we performed gene set enrichment analysis (GSEA) and observed a highly significant enrichment of MYC targets among the upregulated genes (Figure 3B, C, Table S13A), confirming the involvement of c‐MYC in cholangiocarcinogenesis.^63^ Furthermore, the enrichment of E2F and G2M checkpoint genes reflects proliferative activity. Notably, epithelial‐to‐mesenchymal transition (EMT) genes were highly enriched, suggesting that even surgically resectable iCCA can be at an advanced stage harbouring intrinsic alterations such as upregulation of EMT‐inducing transcription factors that might enable the tumour to metastasize.^64^ Downregulated hallmarks included xenobiotic, bile acid, and fatty acid metabolism; coagulation; and adipogenesis, illustrating the contrast between iCCA and the characteristic metabolism of the surrounding liver environment. The pathway enrichments were almost identical when including eight iCCAs for which the transcriptomic data did not meet our quality parameters (Table S13B).

### 3.4 Integrative analysis confirmed known CCA genes and identified new candidates for iCCA carcinogenesis

**FIGURE 4:**
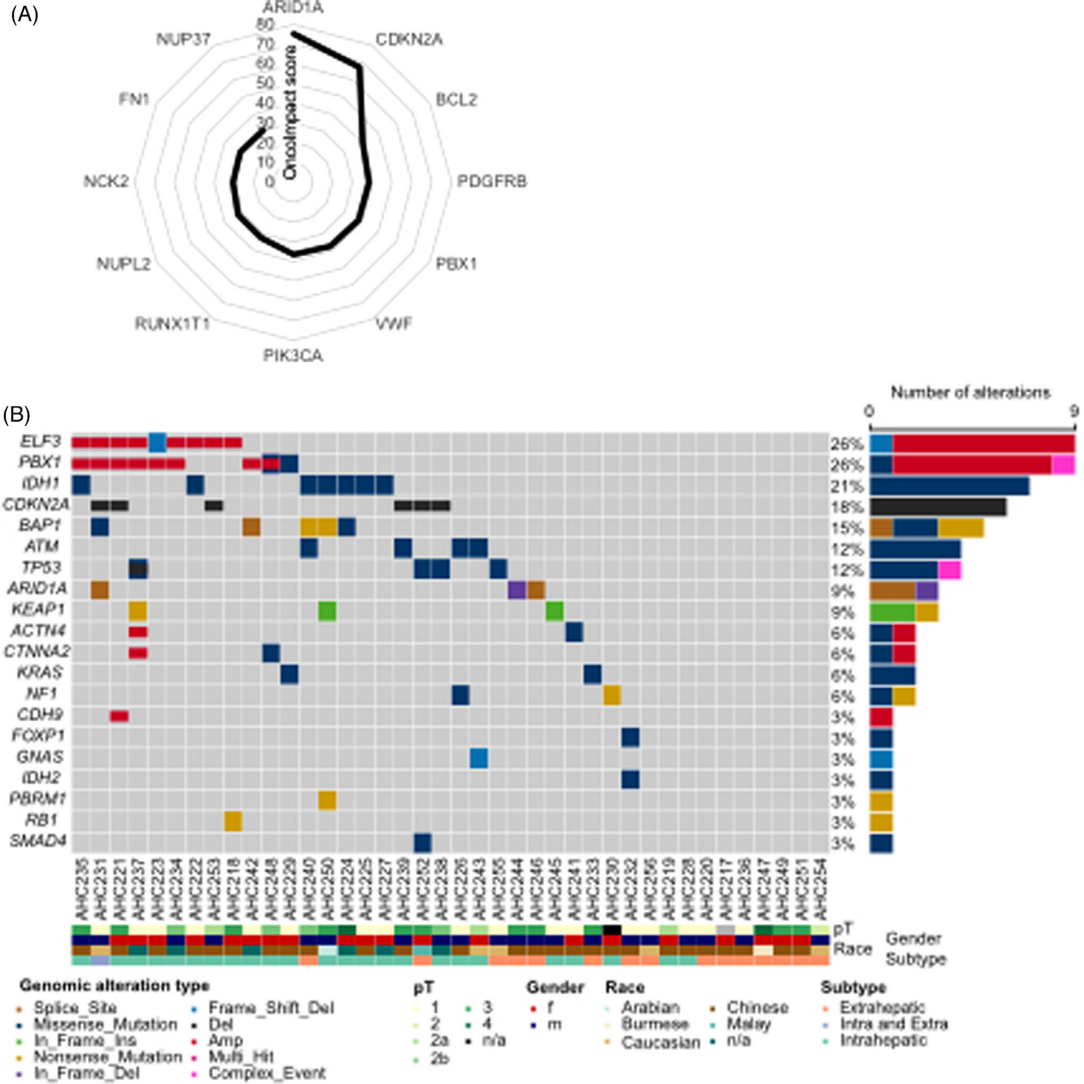
Integrative analysis of the screening cohort data and genomic validation in the second cohort. (A) OncoImpact was used to integrate exome‐wide somatic variant, genome‐wide somatic copy number alteration, and transcriptome‐wide expression data of the screening cohort. The summary statistics of that analysis, the OncoImpact score (OIS), of the top twelve genes are displayed. A complete list of OISs can be found in Table S14. (B) Oncoplot of targeted DNA sequencing of 44 genes in the validation cohort showing the 20 most frequently altered genes with at least one SNV or indel (rows) for 40 CCAs (columns). The tumour stage, sex, tumour subtype and patient ethnic background are indicated at the bottom, and the numbers of somatic alterations per gene are illustrated on the right.

Our main aim was to identify novel genes and pathways that contribute to iCCA development. Single‐gene‐based genomic analyses lack the power to identify new disease‐contributing pathways in situations where different genomic and epigenetic changes can result in the alteration of the same pathway. Therefore, OncoIMPACT was used to identify patient‐specific driver genes by integrative modelling of genomic mutations (SNVs and SCNAs) and the resulting perturbations in transcriptional programs via defined molecular networks.^65^ We identified 100 driver genes that were significantly altered in our cohort of patients with iCCA (Table S14). *ARID1A* had the highest score in this analysis (OncoIMPACT score [OIS] = 75.1), followed by *CDKN2A* (OIS = 67.2; Figure 4A, Table S14). *ARID1A*, which is mostly affected by point mutations, and *CDKN2A* (encoding p16), which is usually altered by deletions, are frequently altered genes in CCA,^23^, ^24^, ^25^, ^26^, ^27^, ^29^, ^66^, ^67^ confirming the validity of our approach. The levels of *ARID1A* and *CDKN2A* OIS were markedly greater than those of the next highest‐ranking genes, *BCL2* (OIS = 40) and *PDGFRB* (OIS = 39). This finding emphasizes the key role of *ARID1A* as an epigenetic modulator within the SWI/SNF complex in iCCA. Loss of expression of SWI/SNF components has been associated with shorter survival in patients with CCA.^68^ Loss of *CDKN2A* function is a common prerequisite for many cancers and is associated with shorter overall survival (OS) in patients with iCCA.^69^ *BCL2* and its family members control apoptosis and play important roles in many cancers.^70^ We observed several BCL2 family members with altered expression in our cohort (Table S13). *PDGFRB* is known to have proproliferative functions in cancer by signalling through the phosphatidylinositol 3 kinase (PI3K) and mitogen‐activated protein kinase (MAPK) pathways.^71^ *PDGFRB* has been reported to be upregulated in cancer‐associated fibroblasts of CCA.^72^ To date, *BCL2* has not been shown to play a prominent role in CCA, and little is known about *PDGFRB* in this context. Initial evidence has been generated thus far, as BCL2‐high iCCAs have been associated with better prognosis, lower pT category and lower frequency of periductal infiltration.^73^ In vivo experiments utilizing the experimental inhibition of PDGFRB via imatinib resulted in reduced tumour growth and increased apoptosis in a rat model of CCA.^74^ Thus, these pathways might be potential targets for iCCA since *PIK3CA*, a central member of the PI3K complex, ranks at position seven according to OncoIMPACT analysis, with an OIS of 36.6.

Interestingly, *PBX1* has not been previously described as relevant for iCCA and was ranked at position five in our integrative analysis (OIS = 38.0). Pre‐B‐cell leukaemia homeobox transcription factor 1 (PBX1) is a transcription factor that regulates numerous embryonic processes including hematopoiesis.^75^ In cancer, *PBX1* was first identified as a fusion gene partner in pre‐B‐cell leukemia.^76^, ^77^ In recent decades, data have shown that *PBX1* contributes to the carcinogenesis of several cancers.^78^ Overall, our integrative analysis confirmed known CCA genes and identified new potential candidates for driving iCCA development.

### 3.5 Validation of recurrently altered genes confirms frequent alterations in *PBX1*

Next, we aimed to validate the recurrence of genomic alterations in an independent collection of 40 CCAs, including intra‐ and extrahepatic CCAs (24 intrahepatic, 15 extrahepatic, 1 intra‐ and extrahepatic) across different ethnic backgrounds but not liver fluke‐associated CCAs (Table S15). We designed an amplicon‐based sequencing panel for genomic DNA targeting the coding regions of 44 genes selected based on recurrent genomic alterations in our German iCCA cohort and prioritization by the literature (Table S16) to identify somatic SNVs and CNAs. Nine samples from the German iCCA cohort were included to validate SCNA calling from amplicon sequencing (Methods and Figure S4).

We observed alterations in *ELF3* in 9 out of 40 CCAs (26%), with copy number gains in 8 out of 9 cases, 7 intrahepatic, 1 intra‐ and extrahepatic (Figure 4B). An *ELF3* mutation frequency of 10.6% has been reported in periampullary tumours with predominantly inactivating frameshift or nonsense mutations.^79^ *ELF3* copy number gains can be found in 2% of iCCAs in a targeted sequencing dataset by Boerner et al.^30^ analyzed through cbioportal.org, but the functional relevance of the copy gain is unclear, particularly since most loss of function mutations have been reported.^80^ *ELF3* mutations have been found to be more frequent in nonliver fluke associated CCA.^25^ *IDH1* was the gene most frequently altered by missense mutations in our validation cohort (7/40, 21%), with 6/7 alterations in intrahepatic tumours. Mutations in *IDH1* are significantly more frequent in nonliver flukes than in liver fluke‐associated CCA,^25^ and our analysis suggested that this is particularly true for iCCA. We detected small somatic mutations at frequencies of 15% and 12% in *BAP1* (12.5% in iCCA), *ATM* (8.3% in iCCA) and *TP53* (8.3% in iCCA), respectively (Figure 4B), confirming their role in CCA. Notably, we detected mutations in *KEAP1* in 9% (3/40) of CCA patients (12.5% in iCCA patients). Lung cancer patients with mutations in *KEAP1* have a particularly poor prognosis^81^; however, the role of *KEAP1* in CCA has not yet been described. Interestingly, we observed nine CCAs with alterations in *PBX1*, seven of which had copy gains, one had a missense mutation, and one had both types of alterations. Interestingly, all seven copy gains occurred in iCCAs (29.2%). Although copy number analysis of amplicon‐based NGS approaches is less robust than other approaches, our validation work (Figure S4) and the high ranking of *PBX1* in the genome‐wide integrative approach (Figure 4A) prompted us to investigate the role of this protein in CCA.

### 3.6 Prognostic effects of PBX1 in European cohorts with iCCA

**FIGURE 5:**
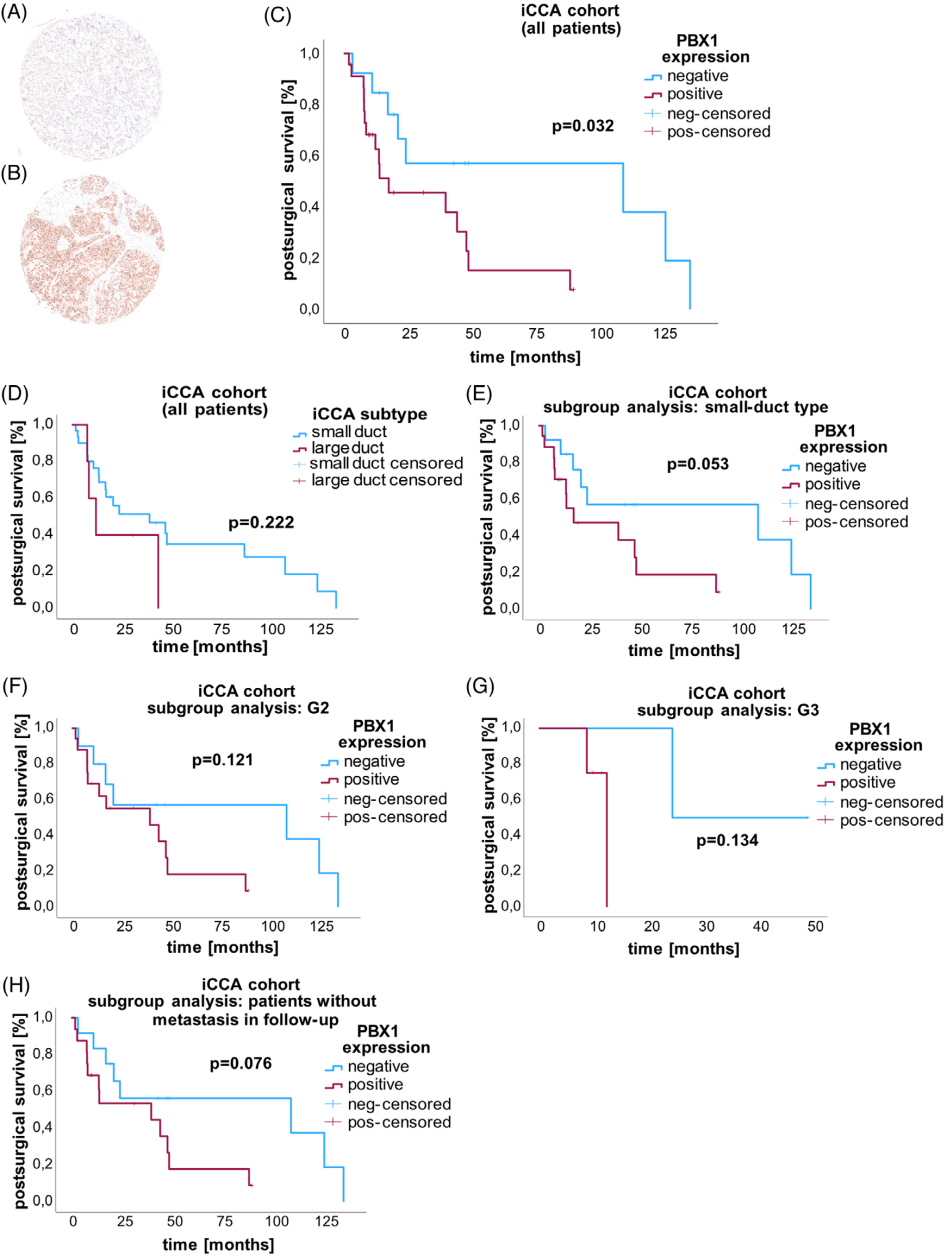
Prognostic impact of PBX1 in an independent European iCCA cohort. Tissue microarrays (TMAs) were generated from an independent German iCCA cohort (A, B) of patients who underwent surgical resection without chemotherapy, and (C) Kaplan–Meier analysis was performed according to PBX1 protein expression at the postoperative follow‐up of 36 iCCAs. TMA spots were immunohistochemically stained and categorized as either (A) samples without PBX1 expression or (B) samples with detectable PBX1. PBX1 expression (*n* = 23) was significantly associated with a poor postoperative prognosis compared with the absence of PBX1 (*n* = 13) (*P* = 0.032). Subgroup analyses (D–I) demonstrated a further negative prognostic impact of PBX1 positivity: large duct‐type iCCA was associated with a worse prognosis (D) (*P* = 0.022), and all patients with this subtype were PBX1 positive (*n* = 5). Among those patients with small duct‐type iCCA (*n* = 31), 18 patients had PBX1 expression and a shorter survival (*P* = 0.053) (E). The G2 (*P* = 0.121) (F) and G3 grades of the tumours (*P* = 0.134) (G) showed similar trends. Only one G1 patient was negative for PBX1 (data not shown). In the subgroup of nonmetastatic patients, a lack of PBX1 expression correlated with improved survival (*P* = 0.076) (H).

To analyze the properties of PBX1, we investigated which transcript is the main mRNA variant and found that *PBX1_202* (ENST00000367897.5, also known as *PBX1b)* was the predominant splice variant in the initial patient cohort as well as in the biliary and CCA cell lines (Figure S5A–C and Supporting information Note). The baseline expression of *PBX1* was the highest in the malignant HuH‐28 cell line, followed by the malignant HuCCT‐1 cell line and was lowest in the nonmalignant MMNK‐1 cell line (Figure S5C). We suppressed and overexpressed *PBX1* in vitro (Figure S5D–F) and observed decreased proliferation in *PBX1*‐overexpressing HuH‐28 cells but also in MMNK‐1 cells with reduced PBX1 expression (Figure S6). *PBX1* did not have typical oncogenic effects on migration, chemoresistance, or colony formation (Supporting information Notes, Figures S7–S9, Table S19, and Supporting information Methods). Although we could not define a specific PBX1‐dependent cell phenotype in the tissue cultures, we evaluated the possible prognostic consequences using a TMA of a well‐characterized European iCCA cohort from the high‐volume centre of Heidelberg.^46^ This cohort of 36 patients who underwent primary resection of nonmetastatic iCCA were immunohistochemically stained for PBX1 (Figure 5A, B). We identified 13 patients who were negative for PBX1 and 23 patients who showed PBX1 expression. Kaplan–Meier analysis revealed a prognostic disadvantage for patients expressing PBX1 (*P* = 0.032) (Figure 5C). The median survival time was 12.48 months (minimum: 2.00 months—maximum: 90.78 months) for PBX1‐positive patients and 24.51 months (minimum: 3.42 months—maximum: 136.84 months) for patients without immunohistochemically detectable PBX1. Table 2 shows the characteristics of the TMA iCCA cohort. All patients with large duct‐type iCCA were positive for PBX1 (*n* = 5). Further subgroup analyses revealed that PBX1‐positivity tended to be associated with unfavourable outcomes in patients with small duct‐type iCCA (*P* = 0.053) and a nonmetastatic postsurgical course (*P* = 0.076) but was not associated with G2/G3 grade (*P* = 0.121 and *P* = 0.134, respectively Figure 5D–H). The association between PBX1 positivity and short survival was supported by an independent iCCA cohort of surgical patients at the University Hospital of Cologne showing a similar trend (*n* = 15, *P* = 0.076, Figure S10, Supporting information Notes and Methods). Overall, analysis of independent German iCCA cohorts demonstrated an association between PBX1 expression and shorter overall survival.

**TABLE 2:** Characteristics of the iCCA cohort from Heidelberg.

	PBX1‐positive N (%)	PBX1‐negative N (%)	*P*‐value
Total cohort	23	13	
**Age (years)**			0.422
Median	58.1	62.3	
Min–Max	33.45–82.26	45.53–80.11	
**Sex**			0.393
Male	9 (39.1)	7 (53.8)	
Female	14 (60.9)	6 (46.2)	
**pN‐category**			0.269
N0	5 (21.7)	3 (23.1)	
N1	7 (30.4)	1 (7.7)	
Nx	11 (47.8)	9 (69.2)	
**pM‐category**			
M0	23 (100)	13 (100)	
**Grading**			0.980
G1	2 (8.7)	1 (7.7)	
G2	17 (73.9)	10 (76.9)	
G3	4 (17.4)	2 (15.4)	
**iCCA subtype**			0.07
Small duct‐type iCCA	18 (78.3)	13 (100)	
Large duct‐type iCCA	5 (21.7)	0 (0)	
**Metastasis (after surgery)**			0.033
Yes	7 (69.6)	12 (92.3)	
No	16 (30.4)		
n/a		1 (7.7)	
**Postsurgical survival (months)**			0.032
Median	12.49	24.51	
Min–max	2.01–90.78	3.41–136.84	

Abbreviations: n/a, not available; min, minimum; max, maximum.

## 4 DISCUSSION

iCCA is a fatal disease with a devastating prognosis because of its resistance to various therapeutic regimens. Even in the era of multimodal therapy, the prognosis has not improved significantly. However, new therapeutic options have recently become available and may revolutionize our current concepts. Recently, constitutively activating gene fusions of *FGFR2* were identified, providing a molecular subgroup for targeted treatment with tyrosine kinase inhibitors.^36^ Mutations in *IDH1/2* result in metabolic and epigenetic reprogramming, and IDH small‐molecule inhibitors are now available, providing new treatment options for iCCA patients with *IDH1* mutations.^33^ Furthermore, there are FDA‐approved targeted treatment options for BRAF^V600E^‐mutant CCA and PD‐1‐targeting therapy for microsatellite unstable CCA currently available^82^, ^83^, ^84^ These developments illustrate the importance of our genomic understanding of CCA. Most genome‐wide data on iCCA are based on studies performed in Asian cohorts and therefore may not be applicable to patients of European descent. Furthermore, most drug targets have been identified based on recurrent mutations, rather than pathway‐based integrative approaches. In 2019, the World Health Organization (WHO) included a novel classification of iCCA subtypes based on these molecular features (in addition to different clinical or histomorphological features, different risk factors, and prognoses).^68^, ^85^, ^86^ Accordingly, iCCAs are divided into small and large duct types, based on their occurrence and origin.^87^, ^88^, ^89^ Small duct iCCAs develop in the hepatic periphery, whereas large duct iCCAs form large intrahepatic bile ducts near the hepatic hilus.^88^, ^90^

In the current study, we focused on the genomic analysis of iCCA in patients with a European background to gain more information on how the disease might differ in this context and whether an integrative approach allows the identification of new molecular components for iCCA. Using comprehensive genomic approaches, we characterized the genomic landscape of European iCCA. We confirmed high mutation frequencies of *ARID1A, IDH1, BAP1, TP53, KRAS*, and *ATM* and observed *CCND1* and *MDM2* in regions with recurrent copy gains, while *FOXP1* and *CDKN2A* were frequently deleted. Four fusion genes were identified. First described in this tumour entity by Neumann et al.^58^ in 2022, we observed the fusion gene *FGFR2::KCTD1* in our screening cohort. To our knowledge, another fusion, *TMEM106B::ROS1*, has not yet been described in iCCA. In particular, *ROS1* fusions are rare in iCCA and have only been reported in a few studies.^35^, ^62^, ^91^, ^92^, ^93^ *TMEM106B::ROS1* has previously been found in non–small cell lung cancer,^59^ and the resulting fusion protein contains a ROS1 tyrosine kinase domain with assumed oncogenic activity. A response to the ROS1 inhibitor crizotinib has been reported in an iCCA patient.^92^ To support the assumption of the oncogenic activity of the *TMEM106B::ROS1* fusion protein, we used a Ba/F3 transformation assay in which IL‐3 was withdrawn from *TMEM106B::ROS1*‐expressing cells. The fact that *TMEM106B::ROS1*‐Ba/F3 cells survived despite the absence of IL‐3 suggests a phenomenon known as “transfer of oncogene addiction”. We found that fusion‐positive cells had a proliferative and survival advantage over cells lacking the fusion gene, providing evidence that *TMEM106B::ROS1* has oncogenic properties.

Integrative genomic/transcriptomic analysis identified *PBX1* (PBX homeobox 1) as a putative new factor in iCCA development. Survival analysis of a German iCCA cohort demonstrated that *PBX1* expression was a negative prognostic marker for iCCA. *PBX1* is located on chromosome 1q23.3 and encodes a transcription factor. First described in 1990^76^, ^77^ as an alternate partner of chromosomal translocation in human pre‐B‐cell acute lymphoblastic leukaemia, several physiological and pathological functions of *PBX1* have been elucidated. It interacts with other cofactors by forming heterodimers with partners such as *HOXB1* or *MEIS1* during transcription in an isoform‐specific manner.^94^, ^95^, ^96^ Approximately 25 transcripts of this gene have been identified and predicted. The most common splicing variants are *PBX1_202* (also known as *PBX1b*, ENST00000367897.5) and *PBX1_203* (also known as *PBX1a*, ENST00000420696.6). Both transcripts regulate the pluripotency regulatory network by influencing stem cell fate. *PBX1_203* appears to stimulate self‐renewal and inhibit differentiation, whereas *PBX1_202* may control cell proliferation and chromosomal accessibility.^97^ Therefore, *PBX1* is reportedly involved in physiological development during embryogenesis and organogenesis.^98^ Nevertheless, dysregulation of these cellular processes can occur in malignant diseases, as *PBX1* has been shown to be altered in several cancer types, including gastric cancer,^99^ lung cancer,^100^ lymphoma,^101^ ovarian cancer,^102^ and esophageal squamous cell carcinoma.^103^ Indeed, there is evidence that *PBX1* is involved in at least five of the major hallmarks of cancer to date: sustaining proliferative signaling, activating invasion and metastasis, inducing angiogenesis, resisting cell death, and deregulating cellular energetics.^78^ In addition, PBX1 has been identified as a pioneer factor that can recognize and bind to specific complementary sequences of DNA, even in highly condensed heterochromatin, thus allowing access to transcriptionally inactive genomic loci by opening chromatin and facilitating the binding of other transcription factors.^104^, ^105^, ^106^, ^107^ However, *PBX1* might also have tumour‐suppressive functions because it can activate the transcription of some DNA damage response genes^78^ and can be suppressed in pediatric acute myeloid leukemia patients.^108^ We did not observe classical oncogenic features in standard tissue culture experiments, suggesting a defining role of cell lineage/cell‐type rather than an oncogenic/tumour suppressor role for PBX1 in CCA.

In summary, our exome‐wide data from a German iCCA cohort provide a resource for a population‐based meta‐analysis of liver fluke‐negative iCCA patients. We identified new fusion genes that extend the list of targetable genomic alterations in iCCA. Finally, we identified *PBX1* as a novel prognostic marker within a network of iCCA alterations. Its expression is associated with short overall survival.

## AUTHOR CONTRIBUTIONS

Ines Gockel, Johannes Schumacher, and Axel M. Hillmer designed the study; Johannes Schumacher and Axel M. Hillmer coordinated the study; Isabelle Morgenstern, Jens U. Marquardt, Michael Heise, René Thieme, Maria Hoppe‐Lotichius, Gerd Otto, Reinhard Buettner, Anja Lautem, Jing Han Hong, Simona O Dima, Irinel Popescu, Steve Rozen, Bin Tean Teh, Hauke Lang, Patrick Tan, and Ines Gockel obtained clinical samples and curated clinical data; Timo Hess, Denis Bertrand, Oscar Velazquez Camacho, Burton Kuan Hui Chia, and Niranjan Nagarajan performed bioinformatics analyses; Christoph Jonas and Christina Alidousty performed fusion gene analysis; Britta Wagner, Stephanie Roessler, Thomas Albrecht, Alexander Quaas, Benjamin Goeppert, and Uta Drebber performed IHC analysis, Timo Hess and Jessica Becker performed Illumina array analyses; Patrick S. Plum, Vanessa Richartz, Barbara Holz, and Sascha Hoppe performed tissue culture experiments; Huay Mei Poh, Cheryl Xueli Chan, Thushangi Pathiraja, Audrey SM Teo, Alexis Khng, and Yao Fei performed NGS sample preparation; Patrick S. Plum, Timo Hess, Denis Bertrand, Oscar Velazquez Camacho, Christoph Jonas, Burton Kuan Hui Chia, Thushangi Pathiraja, Sebastian Klein, Niranjan Nagarajan, and Axel M. Hillmer performed data analyses; Patrick S. Plum, Timo Hess, Denis Bertrand, Thushangi Pathiraja, Niranjan Nagarajan, Johannes Schumacher, and Axel M. Hillmer interpreted the data; and Patrick S. Plum, Timo Hess, and Axel M. Hillmer drafted the manuscript with contributions from Denis Bertrand, Oscar Velazquez Camacho, Christoph Jonas, Christina Alidousty, and Johannes Schumacher.

## CONFLICT OF INTEREST STATEMENT

RB is funded by the German Cancer Aid in the programme “Excellence Center for Oncology‐CIO ABCD”, Center for Molecular Medicine, he has received consulting fees from AbbVie, Amgen, AstraZeneca, Bayer, BMS, Boehringer‐Ingelheim, Illumina, Janssen, Lilly, Merck‐Serono, MSD, Novartis, Qiagen, Pfizer, Roche, Sanofi, Targos MP Inc., he received lecture and presentation honoraries from AbbVie, Amgen, AstraZeneca, Bayer, BMS, Boehringer‐Ingelheim, Illumina, Janssen, Lilly, Merck‐Serono, MSD, Novartis, Qiagen, Pfizer, Roche, Targos MP Inc., he is a member of the board of trustees of the German Cancer Aid and chairs the board of trustees for the Vladimir Totovic Foundation of the German Division Internation Academy of Pathology (GDIAP) and is co‐owner of Timer Therapeutics (Germany) and Gnothis Inc. (Sweden). AMH received research funds from Dracen Pharmaceuticals Inc. and a presentation honorary from AstraZeneca. JUM received honoraria and travel grants from AstraZeneca, Ipsen, MSD, and Roche, which are unrelated to the work presented here. The other authors declare that they have no competing financial interests or personal relationships.

## ETHICAL APPROVAL

All procedures were in accordance with the ethical standards of the responsible committee on human experimentation (institutional and national) and the Helsinki Declaration of 1964 and later versions. Informed consent was obtained from all patients. This study was approved by the ethics committee of the State Medical Association Rhineland‐Palatinate (837.326.08(6323)).

## Supporting information

Supporting Information

Supporting Information

## Data Availability

WES data from the screening cohort and targeted sequencing data from the validation cohort, SNP‐ and expression array data from the screening cohort and RNA‐sequencing data from the cell lines have been deposited at the European Genome‐phenome Archive (EGA), which is hosted by the EBI and the CRG, under accession number EGAS00001007525.
