# Supplementary material for: Integrative genomic analyses of European intrahepatic cholangiocarcinoma: Novel ROS1 fusion gene and PBX1 as prognostic marker
Source: Clin Transl Med. 2024 Jun 14;14(6):e1723. doi: 10.1002/ctm2.1723 (PMC11178519; doi:10.1002/ctm2.1723)
Supplement: Supplementary file 1 — Supporting Information [file CTM2-14-e1723-s001.docx]

**Integrative genomic analyses of European intrahepatic cholangiocarcinoma: Novel *ROS1* fusion gene and PBX1 as prognostic marker**

Patrick S. Plum,^1,2,3,^* Timo Hess,^4,^* Denis Bertrand,^5,^* Isabelle Morgenstern,^6^ Oscar Velazquez Camacho,^2^ Christoph Jonas,^2^ Christina Alidousty^2^, Britta Wagner,^2^ Stephanie Roessler,^7,8^ Thomas Albrecht^7,8^, Jessica Becker,^9^ Vanessa Richartz,^2^ Barbara Holz,^2^ Sascha Hoppe,^2^ Huay Mei Poh,^10^ Burton Kuan Hui Chia,^5^ Cheryl Xueli Chan,^10^ Thushangi Pathiraja,^10^ Audrey SM Teo,^10^ Jens U. Marquardt,^11,12^ Alexis Khng,^10^ Michael Heise,^6,13^ Yao Fei,^10^ René Thieme,^3^ Sebastian Klein,^2^ Jing Han Hong,^14,15^ Simona O Dima,^16^ Irinel Popescu,^16^ Maria Hoppe-Lotichius,^6^ Reinhard Buettner,^2^ Anja Lautem,^6^ Gerd Otto,^17^ Alexander Quaas,^2^ Niranjan Nagarajan,^5^ Steve Rozen,^14^ Bin Tean Teh,^14^ Benjamin Goeppert,^20,21^ Uta Drebber,^2^ Hauke Lang,^6^ Patrick Tan,^14,18^ Ines Gockel,^3^ Johannes Schumacher,^4,#^ Axel M. Hillmer,^2,10,19,#^

1) University of Cologne, Faculty of Medicine and University Hospital Cologne, Department of General, Visceral, Cancer and Transplantation Surgery, Cologne, Germany

2) University of Cologne, Faculty of Medicine and University Hospital Cologne, Institute of Pathology, Cologne, Germany

3) University Hospital of Leipzig, Department of Visceral, Transplant, Thoracic and Vascular Surgery, Leipzig, Germany

4) University Hospital of Marburg, Center for Human Genetics, Marburg, Germany

5) Genome Institute of Singapore, Computational and Systems Biology, Agency for Science, Technology and Research (A*STAR), Singapore, Republic of Singapore

6) Johannes Gutenberg University, General, Visceral and Transplant Surgery, Mainz, Germany

7) Institute of Pathology, University of Heidelberg, Im Neuenheimer Feld 224, 69120 Heidelberg, Germany

8) Liver Cancer Center Heidelberg (LCCH), Heidelberg, Germany

9) Institute of Human Genetics, University Hospital of Bonn, Bonn, Germany

10) Genome Institute of Singapore, Cancer Therapeutics and Stratified Oncology, Agency for Science, Technology and Research (A*STAR), Singapore, Republic of Singapore

11) Johannes Gutenberg University, I Department of Medicine, Mainz, Germany

12) Department of Medicine, University Hospital Schleswig-Holstein, Campus Lübeck, Lübeck

13) Department for General, Visceral and Transplant Surgery, University Hospital Frankfurt, Goethe-University Frankfurt/Main, Frankfurt/Main, Germany

14) Duke-NUS Medical School, Cancer and Stem Cell Biology, Singapore, Republic of Singapore

15) National Cancer Centre Singapore, Division of Medical Science, Laboratory of Cancer Epigenome, Singapore

16) Center of Digestive Diseases and Liver Transplantation, Fundeni Clinical Institute, Bucharest, Romania

17) Emeritus of the Division of Transplantation Surgery, University Medical Center, Langenbeckstr.1, 55131, Mainz, Germany

18) Genome Institute of Singapore, Agency for Science, Technology and Research (A*STAR), Singapore, Republic of Singapore

19) University of Cologne, Center for Molecular Medicine Cologne, Cologne, Germany

20) Institute of Pathology and Neuropathology, RKH Klinikum Ludwigsburg, Ludwigsburg, Germany

21) Institute of Tissue Medicine and Pathology, University of Bern, Bern, Switzerland

**Index**

[Supplementary Notes 2](#_Toc158996060)

[Supplementary Methods 4](#_Toc158996061)

[Supplementary Figures 14](#_Toc158996062)

[Supplementary Tables 25](#_Toc158996063)

### Supplementary Notes

***In-vitro* characterizing malignant and non-malignant biliary cell lines for PBX1**

Having identified PBX1 as a novel putative key player in the pathogenesis of iCCA development, we aimed to analyze its influence on "classical" tumor cell characteristics such as proliferation, invasion, metastasis and chemotherapy resistance to identify a specific *PBX1*-dependent phenotype. To investigate whether these effects are only relevant in malignant cells or can also affect normal bile duct tissue, we decided to compare MMNK-1 as a non-malignant, immortalized biliary cell line [1] with HuH-28 [2] and HuCCT-1 [3] as malignant cholangiolar tumor cell lines (**Supp. Fig. 5a**). Since it was unclear which *PBX1* transcript was predominant in each cell line, quantitative reverse transcription PCR (qRT-PCR) experiments were conducted for the most common splicing variants *PBX1_202 (PBX1b)* (ENST00000367897.5), *PBX1_203* (*PBX1a*) (ENST00000420696.6), and *PBX1_222* (ENST00000699845.1) (reference genome: GRCh38.p12). We observed that *PBX1_202* was the predominant isoform of the transcription factor in all of these cell lines (**Supp. Fig. 5b**). qRT-PCR was then used to examine the differential expression levels between the three cell lines, which revealed lowest expression of *PBX1* for the non-malignant bile duct derived MMNK-1 compared to the malignant iCCA cell lines HuH-28 and HuCCT-1 (**Supp. Fig. 5b**). Interestingly, this was particularly true for the predominant transcript *PBX1_202*, which was expressed 100 to 1000-fold higher in these tumor cells.

To elucidate possible effects of *PBX1*-regulated pathways within the cell, both stable *PBX1* knockdown and overexpression cell lines were generated (**Supp.** **Fig. 5c, d**). Knockdown cell lines from MMNK-1 and HuCCT-1 were established by treatment with several short hairpin -RNAs (sh-RNAs) suppressing all three *PBX1 transcripts (sh4* -> inhibition of PBX1_203 + *PBX1_202* + *PBX1_222; sh9* -> inhibition of PBX1_203 + *PBX1_202* ; sh10 → inhibition of *PBX1_203* + *PBX1_202* + *PBX1_222*). We were not able to generate a viable knockdown cell line for HuH-28, the malignant cell line with the highest intrinsic *PBX1* expression (**Supp. Fig. 5c**) while transfection of MMNK-1 and HuCCT-1 followed by selection resulted in a stable reduction of *PBX1*. On the other hand, the predominant transcript *PBX1_202* was stably overexpressed in all three cell lines (**Supp.** **Fig. 5d**). Again, HuH-28 showed the lowest relative increase in *PBX1* expression compared to the other cell lines. In contrast, the non-malignant MMNK-1 showed the highest relative increase in expression. Western blot analyses demonstrated that PBX1 expression was altered on both, the RNA and protein level.

**Functional aspects of PBX1 in malignant and non-malignant biliary cell lines**

After the successful generation of these *PBX1* knockdown and overexpression cell lines, respectively, we investigated classical cancer-relevant phenotypes of the cell lines. In proliferation assays, suppression of *PBX1* in the benign MMNK-1 cells resulted in a decreased proliferation rate compared to transfection with the corresponding non-coding sh-RNA (**Supp. Fig. 6a**). No such effect was observed in the tumor cell line HuCCT-1 (**Supp. Fig. 6c**). On the other hand, *PBX1* up-regulation in stable transfectants did not affect proliferation in MMNK-1 and HuCCT-1 cells (**Supp.Fig. 6b,d**), whereas HuH-28 showed a significantly reduced proliferation upon *PBX1* overexpression (p<0.005 after 72 hours of growth, **Supp. Fig. 6e**). To evaluate the migration ability of the cells, scratch assays were analyzed. No clear changes were detected depending on the PBX1 expression status in any of the cell lines (**Supp Fig. 7**). Similarly, no strong effects on colony formation or invasion assay upon PBX1 up- or down regulation could be observed (**Supp Fig. 9**). Overall, *PBX1* seems not to have a strong influence on classical cancer hallmarks that are typically tested in tissue culture. It seems possible that *PBX1* has a cell context-dependent influence on proliferation.

Further, we investigated a potential influence of *PBX1* on resistance to the chemotherapeutic drugs currently used to treat CCA such as 5-FU, gemcitabine, cisplatin or capecitabine [4–8]. We did not observe strong effects of *PBX1* overexpression on MMNK-1, HuH-28, and HuCCT-1 cells with a trend to reduced chemoresistance for HuCCT-1 (**Supp. Fig. 8**). The same was true for suppression of *PBX1* in MMNK-1 and HuCCT-1 (**Supp Fig. 8**). Overall, *PBX1* does not have a consistent influence on chemotherapy response.

To understand how PBX1 influences CCA tumor biology, we performed RNA-sequencing analyses of these cell lines to identify downstream genes. Interestingly, despite PBX1 being a transcription factor, we found only a small number of genes with differential expression in PBX1 overexpressing compared to parental cell lines (HuCCT-1: 65 genes, HuH-28: 97 genes, MMNK-1: 1 gene, **Supp. Table 19**). Except for *PBX1*, none of the differentially expressed genes overlapped across cell lines emphasizing again the cell type- and clone-specific effects of *PBX1*. Downregulation of PBX1 resulted in even smaller numbers of differentially expressed genes (HuCCT-1 sh1: 13 genes, HuCCT-1 sh4: 0 genes, MMNK-1 sh4: 0 genes, MMNK-1 sh9: 0 genes, MMNK-1 sh10: 1 gene, **Supp. Table 19**). Only two antisense transcripts overlapped between two analyses, RNF219-AS1 was upregulated in HuH28 PBX1 and down regulated in HuCCT-1 sh1 while PAPPA-AS1 was upregulated in HuCCT-1 PBX1 and down regulated in MMNK-1 sh10. Information on the functional roles of the two antisense RNAs is sparse. Taken together, effects of PBX1 dysregulation seem to be strongly influenced by cell type-specific factors modulating downstream effects.

**Prognostic effects of PBX1 in a monocentric Cologne iCCA cohort**

We analyzed the immunohistochemical expression of PBX1 analogous to the Heidelberg cohort in a TMA from another monocentric, independent cohort from the University Hospital of Cologne. Here, we identified a group of 15 patients (7 female and 8 male) who underwent primary surgical resection due to iCCA. The median age during the time of surgery was 73 years (minimum: 58 years - maximum: 84 years) and the median postsurgical survival was 21 months (minimum: 1 month – maximum: 98 months). PBX1 expression was positive in one patient (6.67%) while 14 patients showed no PBX1 (93.33%). Since the PBX1-positive patient died after the first month after radical surgery, no reliable statistically well powered comparison of prognosis depending on the PBX1-status was available (p=0.076, **Supp Fig. 10)**.

### Supplementary Methods

**Sample Preparation (discovery screen)**

Whole blood and fresh frozen tumor and normal liver tissue samples were obtained from patients undergoing curative surgery at the University Medical Center Mainz, Germany. Genomic DNA isolation from whole blood was performed with the automated Chemagic Magnetic Separation Module I using the Chemagic DNA Blood10k Kit (PerkinElmer) according to the protocol provided by the manufacturer. Tumor and normal liver tissue samples were histopathologically reviewed, macrodissected and subjected to DNA and RNA isolation using the AllPrep DNA/RNA Mini Kit (Qiagen) according to the protocol provided by the manufacturer. RNA quality was further assessed by determining the RNA integrity number (RIN) using the RNA Nano Kit and the Bionanalyzer 2100 (Agilent Technologies) as recommended by the manufacturer. Only RNA samples having a RIN ≥ 6 were taken into consideration for further analyses.

**Whole exome sequencing (WES)**

For exome library preparation 1 µg genomic DNA of tumor/normal sample pairs was sheared into fragments of 100-300 bp size using the S2 Focused-ultransonicator (Covaris). Exome capturing was performed using the SeqCap EZ Human Exome Library V3 (Roche) according to the protocol provided by the manufacturer. The DNA fragments were purified using the Agencourt AMPure XP System (Beckmann Coulter). For end-repair and A-tailing of the DNA fragments the NEBNext End Repair and dA-Tailing Modules (New England Biolabs) were used. For 37 patients, tumor/normal library pairs could be successfully prepared with an additional library for the relapse of patient 26. The libraries were sequenced on a HiSeq 2000 device (Illumina) generating 101 paired-end reads. Further run statistics are provided in **Supp. Table 2**.

Reads were uniquely mapped to the reference genome (GRCh37) using Burrows Wheeler Aligner (BWA). PCR duplicates were removed using PICARD-1.48 (http://picard.sourceforge.net). Genome Analysis Toolkit (GATK-1.0.5974) was used to realign reads near indels and to recalibrate base quality values. Somatic SNV and indel calling was performed using Lofreq somatic (version 2.1.0) and Lofreq indel (version 2.1.0). This list was further filtered by excluding any SNVs/indels called by Lofreq in at least one of the normal controls. Indels were further filtered by a list generated from a relaxed indel calling on the 37 normal samples. The SNVs and indels were annotated using SnpEff (version 3.6).

To evaluate the quality of the variant calls, we performed two tears of validation. Tier I consisted of 47 somatic variants affecting genes categorized as cancer driver genes according to Vogelstein *et al.* [9] that were tested by Sanger sequencing. Out of this set 44 variants could be validated (94%, see blow and **Supp. Table 4**). Two of the three non-validated variants had low allele frequencies of 4 and 5%, respectively, below the detection limit of Sanger sequencing. As tier II, we randomly selected 143 non-silent somatic variants and performed targeted deep sequencing using Illumina technology and validated 136 (95%, see below **Supp. Table 5**), confirming a high validation rate.

**SNV/indel validation by Sanger sequencing**

Primer pairs covering the respective genomic regions of interest were designed using the software Primer3 (v. 4.0.0) and sequence information was obtained from the UCSC Genome Browser (GRCh37). PCR was performed using the HotStarTaq DNA Polymerase (Qiagen) and 20 ng of genomic DNA. PCR cycling parameters included one cycle at 95°C for 15 min, 40 cycles at 94°C for 30s, 58°C for 30s, 72°C for 3min and one cycle at 72°C for 10 min. The amplicons were purified using magnetic beads (Agencourt AmPure, Beckman Coulter) and subjected to bidirectional sequencing using the BigDye Terminator v3.1 Cycle Sequencing Kit (Applied Biosystems). CleanSeq (Agencourt) and a capillary sequencing system (3130XL Genetic Analyzer; Applied Biosystems), as recommended by the manufacturers. Sequences were analyzed using the software SeqMan II of the DNASTAR analysis package (Lasergene, **Supp. Table 4**).

**SNV/indel validation by Illumina deep sequencing**

We designed 136 pairs of primers to target a random subset of 143 SNVs by using Primer3 [10] PCR was carried out with JumpStart^TM^ REDAccuTaq LA DNA Ploymerase (Sigma-Aldrich Inc., St.Louis, MO) in a 50 µl reaction volume with 10 ng of genomic DNA extracted from tumor or matched normal sample as the template. The following program was used: 1) Initial denaturation at 94°C for 3 min, 2) 15 cycles of 20 sec at 94°C, 30 sec at 58°C, 1 min at 68°C, 3) 20 cycles of 20 sec at 94°C, 30 sec at 55°C, 1 min at 68°C, 4) 68°C for 5 min. The PCR product was visualized by agarose gel electrophoresis and the single band at the expected size range was purified by MinElute PCR Purification Kit (QIAGEN). The purified PCR product was eluted into 20 µl nuclease free water, and 1 µl of each purified PCR product generated from tumor or matched normal sample was pooled together. NEBNext**®** DNA Library Prep Master Mix Set for Illumina (New England Biolabs) was used for constructing sequencing libraries using the manufacturer's protocols. Sequencing of tumor and matched normal sample libraries was done using an Illumina MiSeq machine, as pair-end 251 bp reads following the manufacturer's protocols. The reads were mapped to human_g1k_v37. Data is summarized in **Supp. Table 5**.

**Identification and analysis of recurrently mutated genes**

Mutation Annotation Format (MAF) files used to store the detected somatic variants were summarized, analyzed, annotated, and visualized using the maftools Bioconductor package [11]. Only non-silent variants assumed to have an impact on the protein-coding region of these genes were selected. To rank recurrently mutated genes for CCA relevance, all genes having non-silent mutations in at least two tumors were considered. Further, all gene mutations were analyzed by MutSigCV v1.41 ([www.broadinstitute.org/cancer/cga/mutsig](http://www.broadinstitute.org/cancer/cga/mutsig)). Due to limited sample size, nominal P < 0.05 (Fisher exact test) was used to rank genes for relevance for CCA. This gene list was further annotated for entries in the Cancer Gene Census (CGC) database from COSMIC (**Supp. Table 6**).

**Copy number analysis by SNP array**

Tumor and matching control samples were genotyped using the Infinium OmniExpress-24 v1.1 Bead Array (Illumina). 200 ng DNA were used as input and processed according to the standard protocol provided by the manufacturer. Samples achieving a call rate ≥98% in normal tissue samples and ≥90% in tumor samples were included in the subsequent analyses. Normalized total signal intensity (LRR) and B-allele frequency (BAF) values were obtained using the Genotyping module of GenomeStudio (v .1.9.4, Illumina) and subjected to copy number (CN) analysis using ASCAT (v. 2.3, http://heim.ifi.uio.no/bioinf/Projects/ASCAT/). The estimated aberrant cancer cell fraction ranged from 20% to 93%. However, the majority of tumor samples had a tumor fraction equal to or higher than 50% (69.4% of tumor samples). Only samples with an estimated cancer cell fraction ≥ 20% and a goodness of fit score ≥ 80% were taken into consideration for further analyses. For this analysis, the relapse sample CCC-026a and a low tumor content sample CCC-021 were excluded. Copy number data was corrected for tumor ploidy and analyzed for recurrent aberrations using GISTIC 2.0 (https://www.broadinstitute.org/software/cprg/) with default parameters (**Supp. Tables 7-10**). For the OncoImpact analysis only copy number aberrations (CNAs) that were classified as high level amplifications or substantial losses according to the parameters given by the COSMIC database (<http://cancer.sanger.ac.uk/cosmic/analyses>) were used.

**Cell lines and culturing**

The human MMNK-1 (immortalized non-malignant biliary cell line) cell line was kindly provided by Margarete Odenthal (Institute of Pathology, University of Cologne, Germany), while the human iCCA-derived malignant HuH-28 cell line was provided by Axel Hillmer from the Cancer Therapeutics and Stratified Oncology, Genome Institute of Singapore, Singapore. The malignant HuCCT-1 (derived from iCCA) was used with the kind permission of the JCRB Cell Bank (Japanese Collection of Research Bioresources Cell Bank) (Japan). Phoenix Eco cell line (CRL-3214 ™) was obtained from ATCC and maintained in DMEM medium (Gibco from Thermo Fisher Scientific Inc., Waltham, MA) supplemented with 10% fetal bovine serum (Cat. No.: P40-37500, Pan Biotech, Aidenbach, Germany), 1% penicillin-streptomycin (Cat. No.: P0781, Sigma-Aldrich, St. Louis, MO), 1% L-glutamine (Cat. No.: P04-08100, Pan Biotech) and 1 mM Sodium Pyruvate (Cat. No.: 11360070, Life Technologies GmbH). Ba/F3 cells were kindly provided by Dr. Johannes Brägelmann (University of Cologne, Germany) and grown in the presence of (2 ng/ml) IL-3 (Cat. No.: 213-13-10, PeproTech GmbH). Both tumor cell lines, HuH-28 and HuCCT-1, as well as Ba/F3 cells were maintained in RPMI 1640 + GlutaMAX™ medium (Gibco from Thermo Fisher Scientific Inc., Waltham, MA) supplemented with 10% fetal bovine serum (Cat. No.: P40-37500, Pan Biotech, Aidenbach, Germany) and 1% penicillin-streptomycin (Cat. No.: P0781, Sigma-Aldrich, St. Louis, MO). MMNK-1 were cultured in DMEM medium (Gibco from Thermo Fisher Scientific Inc., Waltham, MA) supplemented with 10% fetal bovine serum (Cat. No.: P40-37500, Pan Biotech, Aidenbach, Germany), 1% penicillin-streptomycin (Cat. No.: P0781, Sigma-Aldrich, St. Louis, MO), and 1% L-glutamine (Cat. No.: P04-08100, Pan Biotech). All cells were cultured at 37°C in a humidified chamber supplemented with 5% CO2.

**Generation of stable *PBX1* knockdown and *PBX1* overexpression cell lines via transfection and viral transduction**

For *PBX1* knockdown, we used the respective shRNAs (sh4, sh9, sh10). Additionally, a modified non-target (non-coding) shRNA was transfected as a control. The clone IDs and sequences of the shRNAs were as follows: sh4 (TRCN0000274137; target sequence: ATGATCCTGCGTTCCCGATTT), sh9 (TRCN0000274077; target sequence: CGAAGCAATCAGCAAACACACAA), and sh10 (TRCN0000274085; target sequence: TTCAAGAGGAAGCCAATATTT). MISSION® TRC2 pLKO.5-puro Non-Target shRNA Control Plasmid DNA (non-coding) was used as a control for the following experiments. All shRNAs were obtained from Merck (Burlington, USA).

For *PBX1* overexpression, the Lenti ORF clone of human pre-B-cell leukemia homeobox 1 (PBX1) (transcript variant 1) from Origene (Rockville, USA) (CAT#: RC210944L3) was utilized, while the pLenti-C-Myc-DDK-P2A-Puro lentiviral gene expression vector (Origene; Rockville, USA) (CAT#: PS100092) served as a control.

HEKT293T cells were kindly provided by Yue Zhao (Department of General, Visceral, Cancer and Transplant Surgery, University of Cologne, Germany) and were cultured in DMEM medium (Gibco from Thermo Fisher Scientific Inc, Waltham, MA) supplemented with 10% fetal bovine serum (Cat. No.: P40-37500, Pan Biotech, Aidenbach, Germany), 1% penicillin-streptomycin (Cat. No.: P0781, Sigma-Aldrich, St. Louis, MO), and 1% L-glutamine (Cat. No.: P04-08100, Pan Biotech). For transfection, cells were plated in 6-well plates and transfected at 80% density 24 hours after plating using FuGENE HD transfection reagent (Roche, Germany) according to the manufacturer's protocol. Plasmids psPAX2 (RRID: Addgene_12260), MD2.G (RRID: Addgene_12259) were used for viral transduction. Lentiviruses were harvested after 48 hours and used for transfections. The target cells (MMNK-1, HuH-28, and HuCCT-1) were then incubated with the virus-containing supernatant twice for 24 hours (with 24 hours of regeneration in between) before selection with puromycin. After two weeks, the cells were cultured in normal media and used for the following experiments.

Altered PBX1 expression was detected by qRT-PCR and Western Blot to ensure that both RNA and protein levels were altered by the treatment. For Western Blot, cells were cultured in 6-well plates and harvested after 24 hours at 80% density in 500µl Laemmli buffer (ThermoFisher Scientific). Samples were then heated at 98°C for 5 minutes and loaded onto Mini-PROTEAN® TGX^TM^ Gel (12%, 15-well, 15µl) (Bio-Rad, Munich, Germany). Membrane transfer was performed at 100 volts for 40 minutes. This was followed by incubation with blocking buffer (TBST with 5% BSA) and primary antibody (Pbx1 antibody #4342, Cell Signaling); dilution: 1:1000 in TBST + 5% BSA for 60 minutes each at room temperature. After three 5-minute washes in TBST, secondary antibody (anti-rabbit) was applied according to the manufacturer's protocol. Membranes were incubated with Lumi-LightPLUS Western Blot Substrate (Merck, Darmstadt, Germany) and digital images were captured. By stripping with Restore™ Western Blot Stripping Buffer (ThermoFisher Scientific), the antibodies were stripped from the membrane and after blocking with TBST + 5% BSA for 60 minutes, staining was performed with the control antibody against the housekeeping protein GAPDH (GAPDH (6C5): sc-32233, Santa Cruz Biotechnology); dilution: 1:200 in TBST + 5% BSA. Incubation with a secondary anti-mouse antibody took place according to the manufacturer’s protocol.

For further investigation which splicing variant of PBX1 is predominant within the different biliary cell lines and evaluation of the success of both knockdown and overexpression experiments as well as for transcriptomic analyses, mRNA-levels of PBX1 (variants) were detected via real-time PCR (RT-PCR) experiments. Therefore, cells were plated into 6-well plates (300,000 cells per plate) and cultured for 48 hours. Afterwards, harvesting and RNA extraction was performed with the Qiagen AllPrep DNA/RNA Kit (Hilden, Germany) according to the manufacturer’s protocol. Isolated RNA was reverse transcribed into cDNA via Omniscript RT Kit (Qiagen, Hilden, Germany) following the manual. The concentration of cDNA and RNA was determined on a Qubit 3.0 Fluorometer (ThermoFisher Scientific), using the dsDNA HS (High Sensitivity) and RNA BR (Broad-Range) Assay kits, respectively.

RT-PCR was performed using SYBR Green PCR Master Mix (ThermoFisher Scientific), Bio-Rad CFX384 Touch (Bio-Rad, Munich, Germany), and Bio-Rad CFX Manager v3.0 software. Each cDNA sample was measured in triplicate, and ΔCT values of the gene of interest were normalized to the housekeeping gene hypoxanthine phosphoribosyltransferase 1 (HRPT1). The following primers were used to detect the different PBX1 variants HPRT1 (forward) GACCAGTCAACAGGGGACAT, (reverse) GTGTCAATTATATCTTCCACAATCAAG; PBX1_202 (forward) AGCCAAGAAGTGTGGCATCA, (reverse) GGGTATCCACCAGCCGAGTTG; PBX1_203 (forward) ACTCGGCTGGTTCTTCCAG, (reverse) CAACCTCCATTAGCACTGATGC; PBX1_222 (forward) GGGCATCAGTCACCTCCCCC, (reverse) GGGAAATTCGCTGTCGGGGAAAATG; and PBX1_common (forward) GGAGCTGGAGAAATACGAGCA, (reverse) TCCGCTCAATCTCCTTTGGG. Cycler settings were: 40 cycles per run, annealing temperature 59°C (HRPT1), 61°C (PBX1_common), or 62°C (PBX1_202, PBX1_203, and PBX1_222).

**Proliferation, migration, and chemoresistance experiments *in-vitro***

Cell viability assays were performed using the RealTime-Glo™ MT Cell Viability Assay according to the manufacturer’s protocol (Promega, Madison, WI, USA). Therefore, cells were plated in black-walled 96-well plates at a density of 5.000 cells/well (MMNK-1, HuCCT-1) or 10.000 cells/well (HuH-28). Cells were allowed to attach overnight. For time-zero measurements, cells were incubated with RealTime-Glo™ MT Cell Viability reagent for 1 hour at 37 °C, and luminescence was measured on a Synergy (Biotek, Winooski, VT, USA) microplate reader. Luminescence was then measured at 24, 48, and 72 hours after the addition of RealTime-Glo™ reagent.

Scratch assays were performed to determine alternating migration ability. Cells were plated in 6-well plates at a density of 300,000 cells/well and incubated overnight to form a fusion monolayer. A linear scratch was made in the center of each well using a 10 µl plastic pipette tip. After three washes with phosphate-buffered saline, the cells were cultured in serum-free medium. At 0, 12, and 24 hours after scratching, the wounded areas were observed under an inverted microscope. Photographs were taken. Image analysis was performed using ImageJ (National Institutes of Health, Bethesda, USA) to objectify the evaluation. For this purpose, the measured free residual areas were normalized to the area of the initial scratch.

For the soft agar colony formation assay, 100,000 cells per well were inoculated into a base layer containing 1% agar (Micro agar, Cat. No. M1002.1; Duchefa Biochemie B.V.) and a top layer containing 0.7% agar (Agarose SPI, Cat. No. A1203.01; Duchefa Biochemie B.V.). Medium was changed three times/week and 6-well plates were incubated for 14 days at 37°C under 5% CO2. Colonies were defined as >30 cells, counted manually and observed by light microscopy.

Investigation of the influence of PBX1 on invasiveness was performed via invasion assay. Clones of the cell lines were grown on Matrigel in tissue culture inserts with 8 µm pore size in media without fetal calf serum, separated from media containing fetal calf serum. After 24 hours, cells that had invaded through the matrigel and insert pores to the bottom of the inserts were stained with crystal violet, scanned and counted using an automated cell detection system, followed by exclusion of too large and too small particles in R. Three independent experiments were assayed in quadruplicates for each condition.

Next, chemoresistance was analyzed as a function of PBX1 status. Therefore, cells were incubated with increasing concentrations of the following chemotherapeutic agents: 5-fluorouracil (5-FU), cisplatin, gemcitabine and capecitabine. The drugs were supplied by the pharmacy of the University Hospital of Cologne and were diluted in medium to achieve tenfold concentrations between 100 µM and 0.0001 µM. Cell viability was assessed using the WST-1 reagent (Roche Diagnostics, Basel, Switzerland) according to the manufacturer's instructions. For these experiments, cells were plated in 96-well plates at a density of 2,000 cells/well and incubated for 24 hours. The cells were then treated with different concentrations of the four chemotherapeutic agents for an additional 48 hours, and the medium was changed to a phenol red-free medium containing 10% WST-1 reagent. After 2.5 hours of incubation, absorbance was measured at 450 nm wavelength using a Synergy HT Microplate Reader (Bio-Tek Instruments, Winooski, VT, USA).

Unless otherwise noted, all of the above experiments were performed in triplicate in three independent experiments.

**RNA-seq of PBX1 knock-down or overexpressing cell lines**

RNA has been extracted from tissue culture using the AllPrep DNA/RNA Mini Kit (Qiagen) according to the manufacturer's recommendations. One µg of total RNA was used for TruSeq stranded mRNA library preparation (Illumina) according to the manufacturer’s recommendations. Libraries were sequenced on a NovaSeq6000 (Illumina) by 2x150 bases targeting 30 million reads per sample. Paired-end reads were aligned to the hg19 human genome assembly using STAR v. 2.6 [12]. Mapped reads were counted with HTSeq and differential gene expression analysis was conducted using the DESeq2 Bioconductor package version 1.22.2 [13]. An adjusted p-value threshold of 0.05 and a log2 fold change ≥1 were set to determine differential gene expression among all paired-wise comparisons.

**Tissue microarray (TMA) immunohistochemistry from the monocentric Cologne iCCA cohort**

To evaluate the putative prognostic effects of PBX1 in patients of European descent, we performed TMA immunohistochemistry in a cohort of iCCA patients who underwent radical surgical resection in curative intention between 2000 and 2019 at the surgical department of the University Hospital of Cologne, Germany. For standardization, only patients without previous systemic therapy prior to surgery and with a postsurgical survival of at least 14 days were included. In total, 15 patients remained for further analysis. TMA preparation and staining was performed as described for the Heidelberg iCCA cohort assessing a two-tier scoring system for PBX1.

**Funding**

This study was supported by the Agency for Science Technology and Research (A*STAR) Singapore, Marga und Walter Boll-Foundation with grant No. 210-05.02-18 (to PSP), German Research Foundation (DFG) with grants 418074181 and 446411360, and Federal Ministry of Education and Research (BMBF) with grant CompL DeepInsight 031L0267B (to AMH). JUM was supported by a grant from Wilhelm Sander Foundation (2021.089.1).

**References**

1. Maruyama M, Kobayashi N, Westerman KA, et al (2004) Establishment of a highly differentiated immortalized human cholangiocyte cell line with SV40T and hTERT. Transplantation 77:446–451. https://doi.org/10.1097/01.TP.0000110292.73873.25

2. Kusaka Y, Tokiwa T, Sato J (1988) Establishment and characterization of a cell line from a human cholangiocellular carcinoma. Res Exp Med (Berl) 188:367–75. https://doi.org/10.1007/BF01851205

3. Miyagiwa M, Ichida T, Tokiwa T, et al (1989) A new human cholangiocellular carcinoma cell line (HuCC-T1) producing carbohydrate antigen 19/9 in serum-free medium. In Vitro Cell Dev Biol 25:503–10. https://doi.org/10.1007/BF02623562

4. Walter T, Horgan AM, McNamara M, et al (2013) Feasibility and benefits of second-line chemotherapy in advanced biliary tract cancer: a large retrospective study. Eur J Cancer 49:329–35. https://doi.org/10.1016/j.ejca.2012.08.003

5. Park J-S, Oh S-Y, Kim S-H, et al (2005) Single-agent gemcitabine in the treatment of advanced biliary tract cancers: a phase II study. Jpn J Clin Oncol 35:68–73. https://doi.org/10.1093/jjco/hyi021

6. Giuliani F, Gebbia V, Maiello E, et al (2006) Gemcitabine and cisplatin for inoperable and/or metastatic biliary tree carcinomas: a multicenter phase II study of the Gruppo Oncologico dell’Italia Meridionale (GOIM). Ann Oncol Off J Eur Soc Med Oncol 17 Suppl 7:vii73-7. https://doi.org/10.1093/annonc/mdl956

7. Valle J, Wasan H, Palmer DH, et al (2010) Cisplatin plus gemcitabine versus gemcitabine for biliary tract cancer. N Engl J Med 362:1273–81. https://doi.org/10.1056/NEJMoa0908721

8. Primrose JN, Fox RP, Palmer DH, et al (2019) Capecitabine compared with observation in resected biliary tract cancer (BILCAP): a randomised, controlled, multicentre, phase 3 study. Lancet Oncol 20:663–673. https://doi.org/10.1016/S1470-2045(18)30915-X

9. Vogelstein B, Papadopoulos N, Velculescu VE, et al (2013) Cancer genome landscapes. Science (80-. ). 340:1546–1558

10. Untergasser A, Cutcutache I, Koressaar T, et al (2012) Primer3--new capabilities and interfaces. Nucleic Acids Res 40:e115. https://doi.org/10.1093/nar/gks596

11. Mayakonda A, Lin D-C, Assenov Y, et al (2018) Maftools: efficient and comprehensive analysis of somatic variants in cancer. Genome Res 28:1747–1756. https://doi.org/10.1101/gr.239244.118

12. Dobin A, Davis CA, Schlesinger F, et al (2013) STAR: ultrafast universal RNA-seq aligner. Bioinformatics 29:15–21. https://doi.org/10.1093/bioinformatics/bts635

13. Love MI, Huber W, Anders S (2014) Moderated estimation of fold change and dispersion for RNA-seq data with DESeq2. Genome Biol 15:550. https://doi.org/10.1186/s13059-014-0550-8

### Supplementary Figures


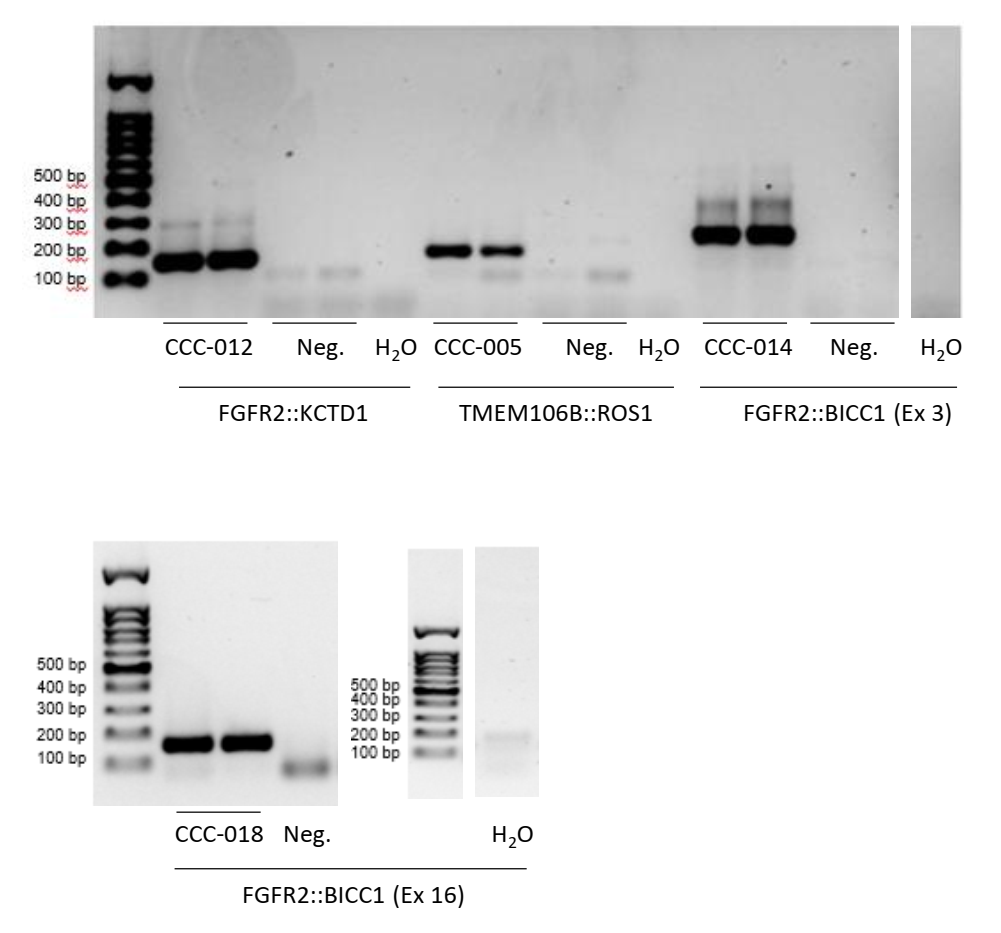


**Supplementary Figure 1: PCR validation of fusion genes.** PCR primer were designed in flanking exons based on targeted RNA-seq (Archer)-derived fusion points to result in 150 to 250 bp amplicons. Investigated fusion genes are indicated at the bottom. Tumor RNA of patients where fusions were identified are indicated with their respective CCC-IDs and fusion-negative tumor RNAs were used as negative controls (Neg.). H2O indicates reactions without cDNA. 100 bp ladder is shown on the left.


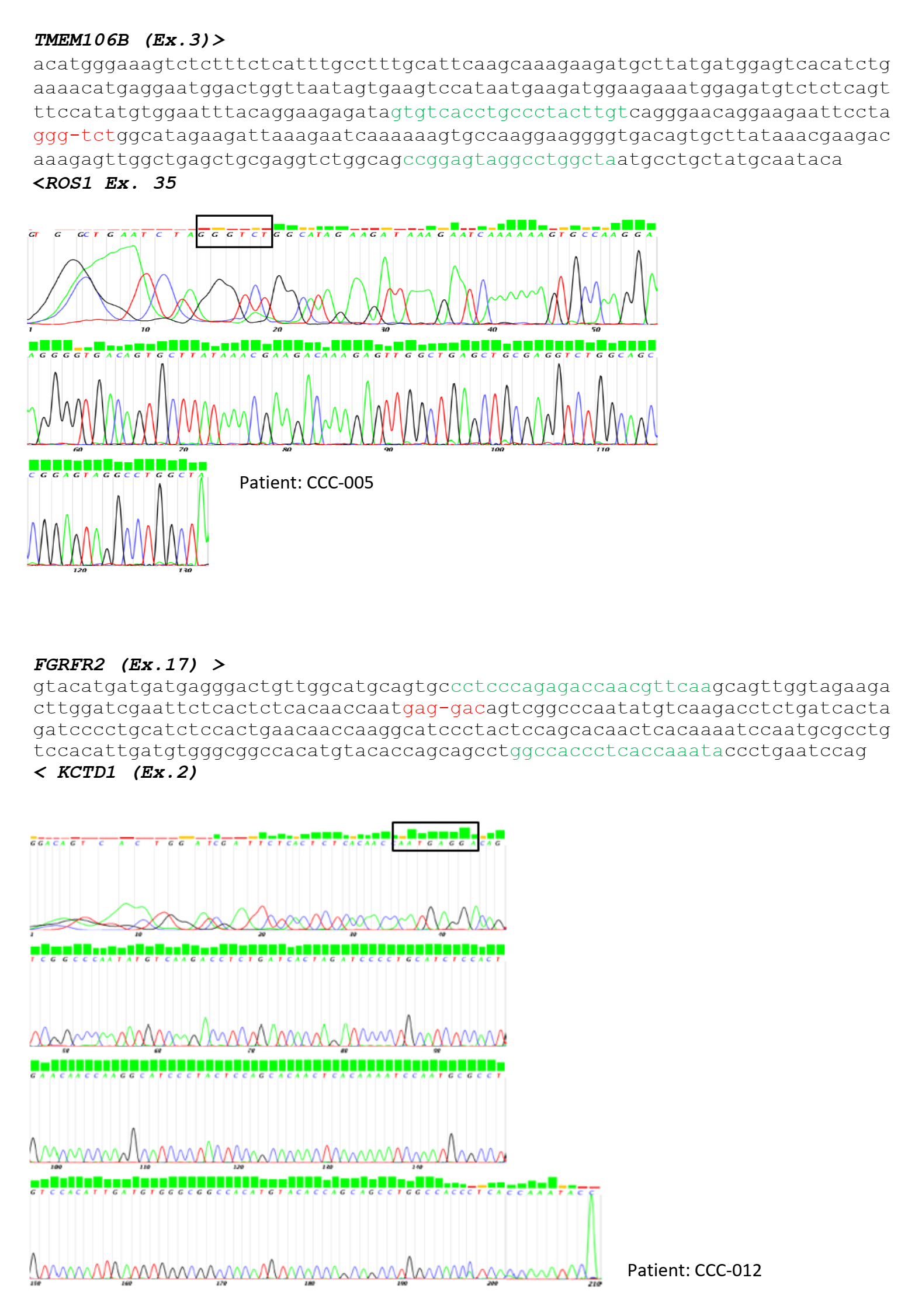


**Supplementary Figure 2: Validation of fusion points by Sanger sequencing.** PCR Products shown in Supp. Fig. 1 are sequenced by Sanger method. Fusion points are indicated by black rectangles in chromatograms (bottom). Derived sequences are shown in their transcript context on top with primers indicated in green and fusion points in red.


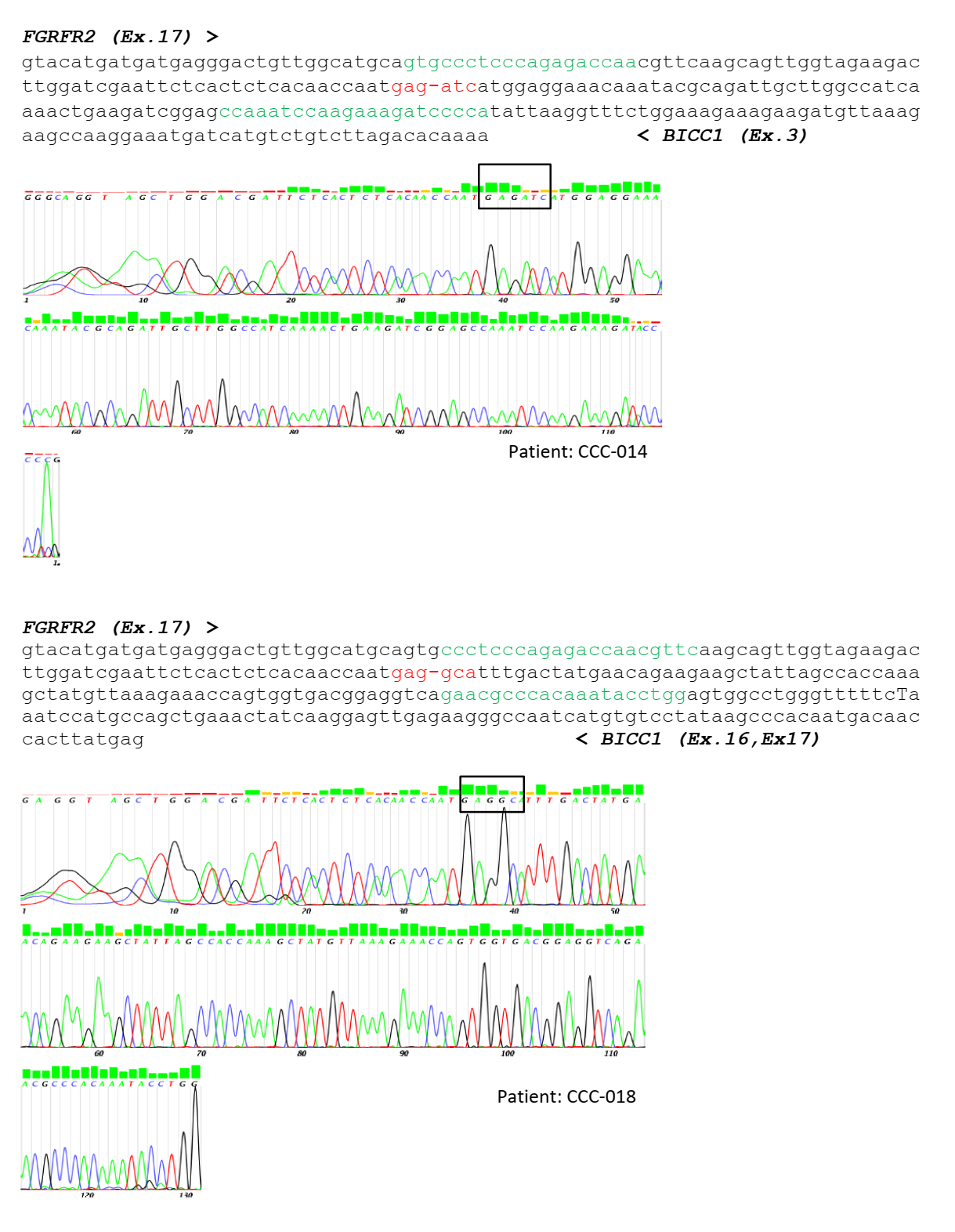


**Supplementary Figure 3: Validation of fusion points by Sanger sequencing.** PCR Products shown in Supp. Fig. 1 are sequenced by Sanger method. Fusion points are indicated by black rectangles in chromatograms (bottom). Derived sequences are shown in their transcript context on top with primers indicated in green and fusion points in red.


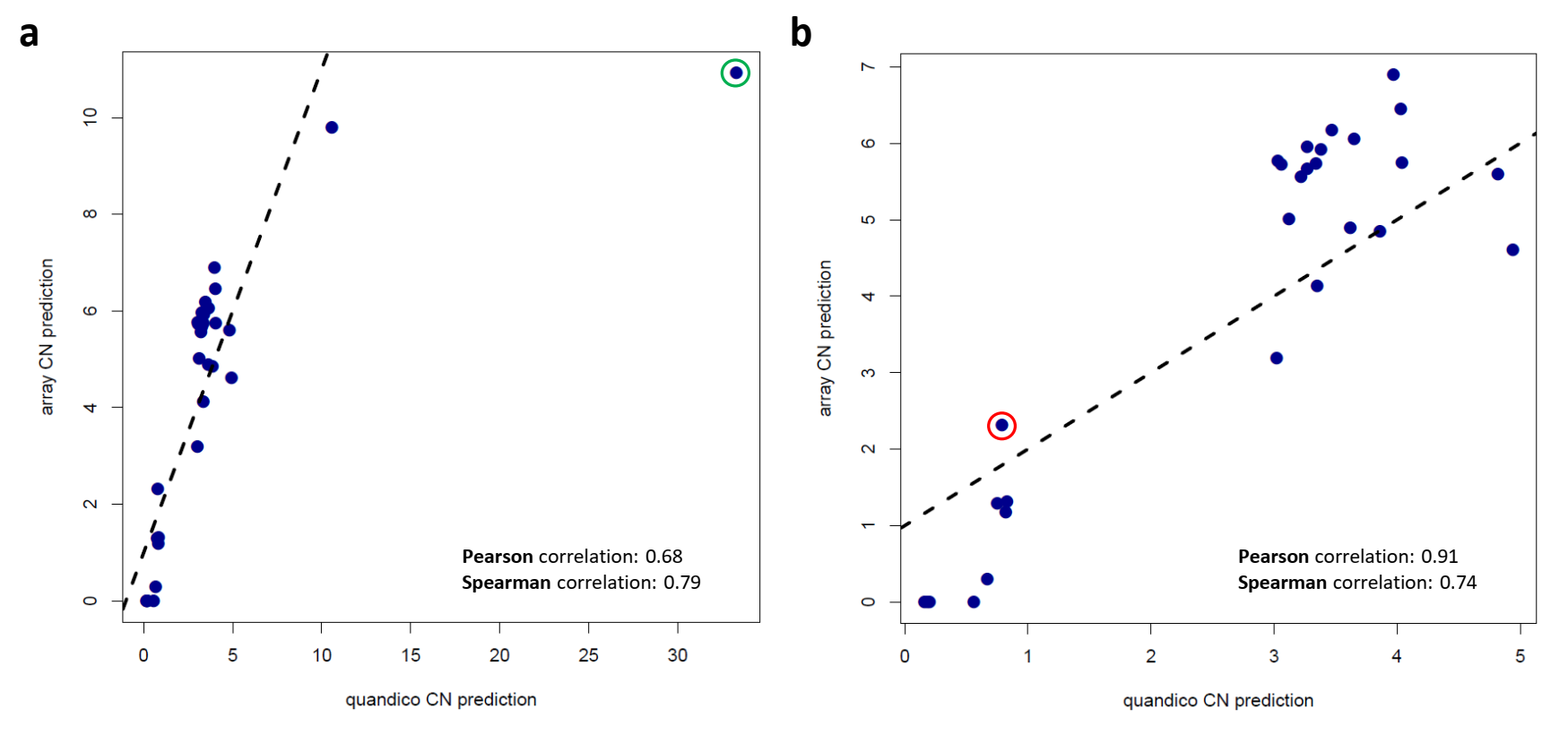


**Supplementary Figure 4: Comparison of array and amplicon sequencing copy number estimation.** Copy number estimates of nine German iCCAs based on SNP array (y axis) were compared with copy number estimates based on targeted sequencing using quandico (x axis). (**a**) All gene-based copy gains and losses affecting any of the 44 genes of the targeted sequencing are plotted for both data sources. Outlier is highlighted in red. It is possible that extremely high copy number values are not captures equally well by the two approaches. Dashed line represents regression line. (**b**) All gene-based copy gains and losses are shown after exclusion of the outlier in (a). Copy number gains and losses are in agreement except one unmatched deletion call for *CDKN2A* in the amplicon-based sequencing analysis. Overall, there is good agreement for the copy number calls across the two different technologies.


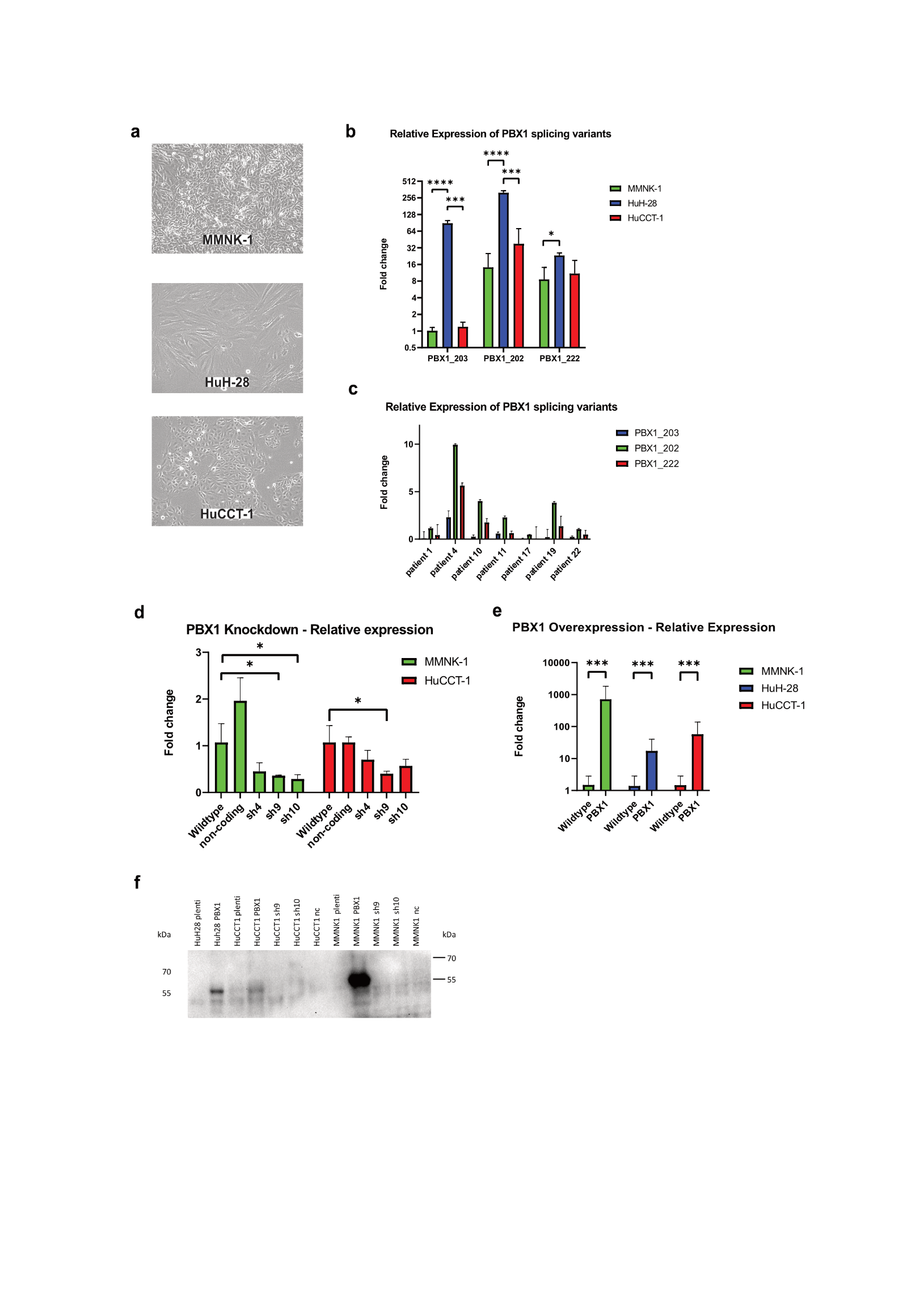


**Supplementary Figure 5**. **Characterization of non-malignant and malignant bile duct cell lines for PBX1.** (**a**) Microscopic images of the non-malignant immortal bile duct cell line MMNK-1 and malignant cholangiolar tumor cell lines HuH-28 and HuCCT-1 derived from human iCCA. (**b**) Comparison of the expression levels of the different PBX1 splice variants relative to the expression of PBX1_203 in MMNK-1 demonstrated the lowest *PBX1*-levels within the non-malignant MMNK-1 cells while the neoplastic HuH-28 and HuCCT-1 cells showed higher expression rates especially for the predominant PBX1_202 transcript. (**c**) PBX1-expression was analyzed for representative patients of the initial German iCCA discovery cohort to evaluate distribution of the different splicing variants. In agreement with *in vitro* data, PBX1_202 was the predominant splicing variant in those patients (**d**) Stable cell lines with different sh-RNAs reducing the expression of *PBX1* resulted in stable *PBX1* knockdown for MMNK-1 and HuCCT-1. For the high *PBX1*-expressing HuH-28 no viable knockdown cell line could be established. (**e**) *PBX1* qRT-PCR for stable *PBX1*(_202) overexpressing cell lines is shown. (**f**) Western blot analysis demonstrated PBX1 protein upregulation for PBX1 overexpressing iCCA cell lines.

Three independent experiments were performed in triplicate (*p<0.05; ***p<0.01, ****p<0.001).


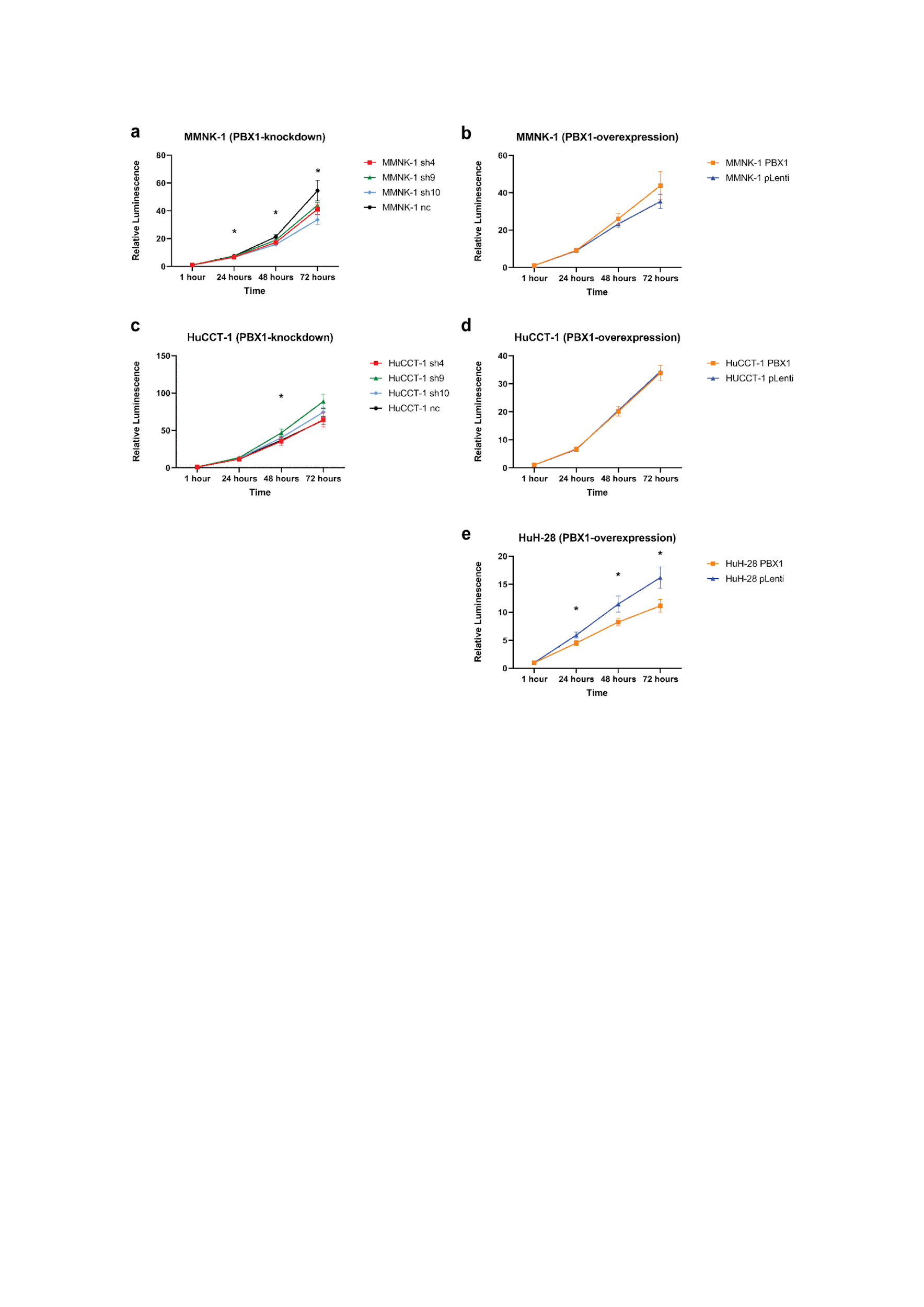


**Supplementary Figure 6.** **Proliferation rates depending on the *PBX1*-expressions in non-malignant and malignant bile duct cell lines.** Real-time Glow assays were performed for *PBX1* knockdown (sh4, sh9, sh10) and *PBX1* overexpressing (PBX1) cell lines compared to the corresponding controls (nc and pLenti, respectively). In benign MMNK-1 cells, *PBX1* reduction (**a**) resulted in decreased proliferation, whereas higher *PBX1* levels (**b**) were associated with increased (non-significant) proliferative capacity. For HuCCT-1, no significant changes in proliferation were observed (**c-d**). *PBX1* overexpression in HuH-28 significantly increased proliferation (**e**). *PBX1* knockdown did not generate viable cell clones for HuH-28 and therefore no analyses could be performed. Three independent experiments were performed in triplicate (*p<0.05)


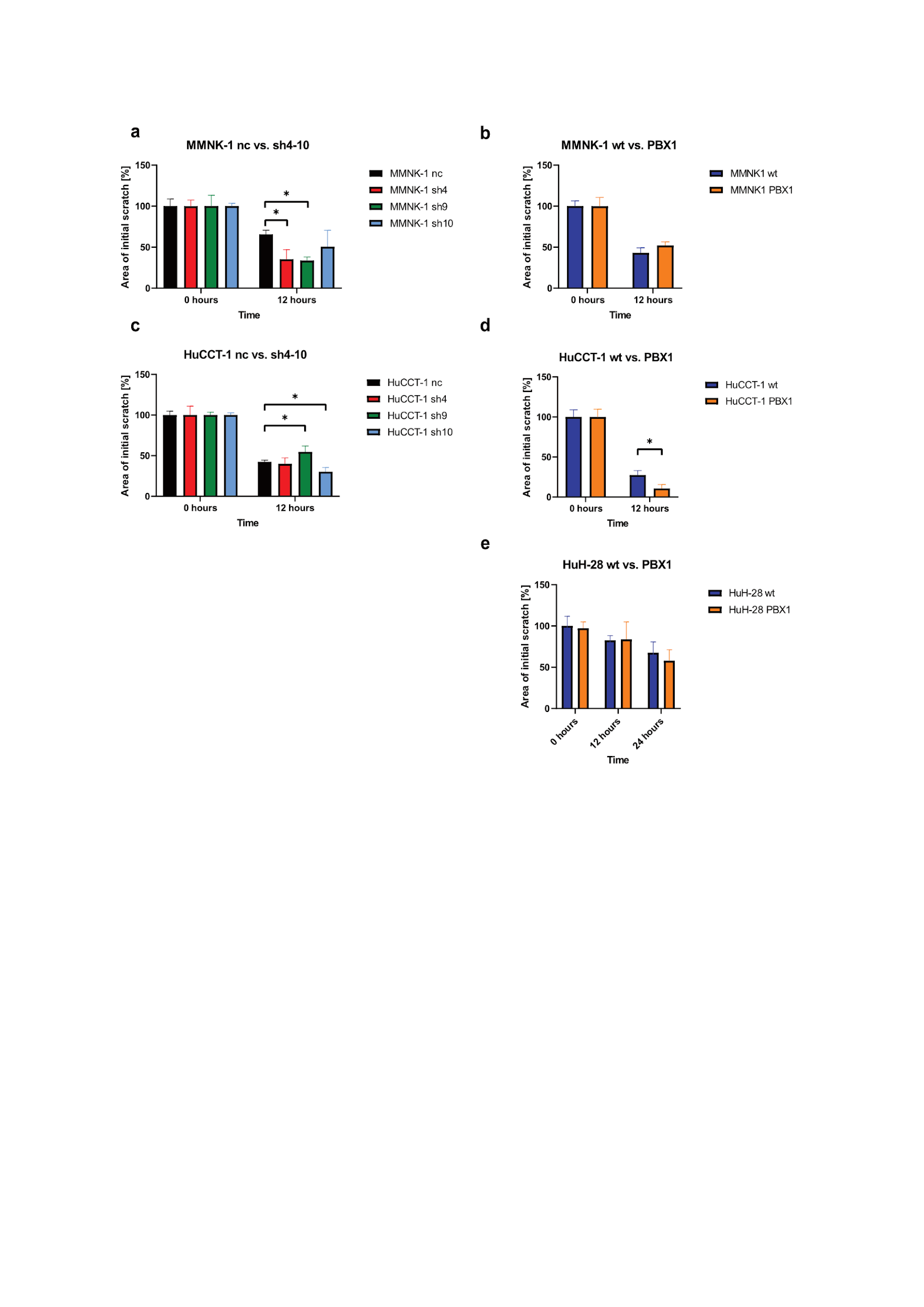


**Supplementary Figure 7.** **Migration ability as a function of PBX1 expression in non-malignant and malignant bile duct cell lines.** Wound healing assays were performed for *PBX1* knockdown (sh4, sh9, sh10) and *PBX1* overexpressing (PBX1) cell lines compared to the corresponding controls (nc (non-coding) and wt (wild-type), respectively). In both viable *PBX1* knockdown cell lines (MMNK-1 and HuCCT-1), no clear *PBX1*-associated phenotype was detected (**a, c**). While in MMMK-1 elevated *PBX1* levels did not affect cell migration (**b**), *PBX1* overexpression positively correlated with this ability in HuCCT-1 (**d**). In HuH-28 with high intrinsic *PBX1* activity, further increase in *PBX1* did not result in alternate migration (**e**). Three independent experiments were performed in triplicate (*p<0.05).


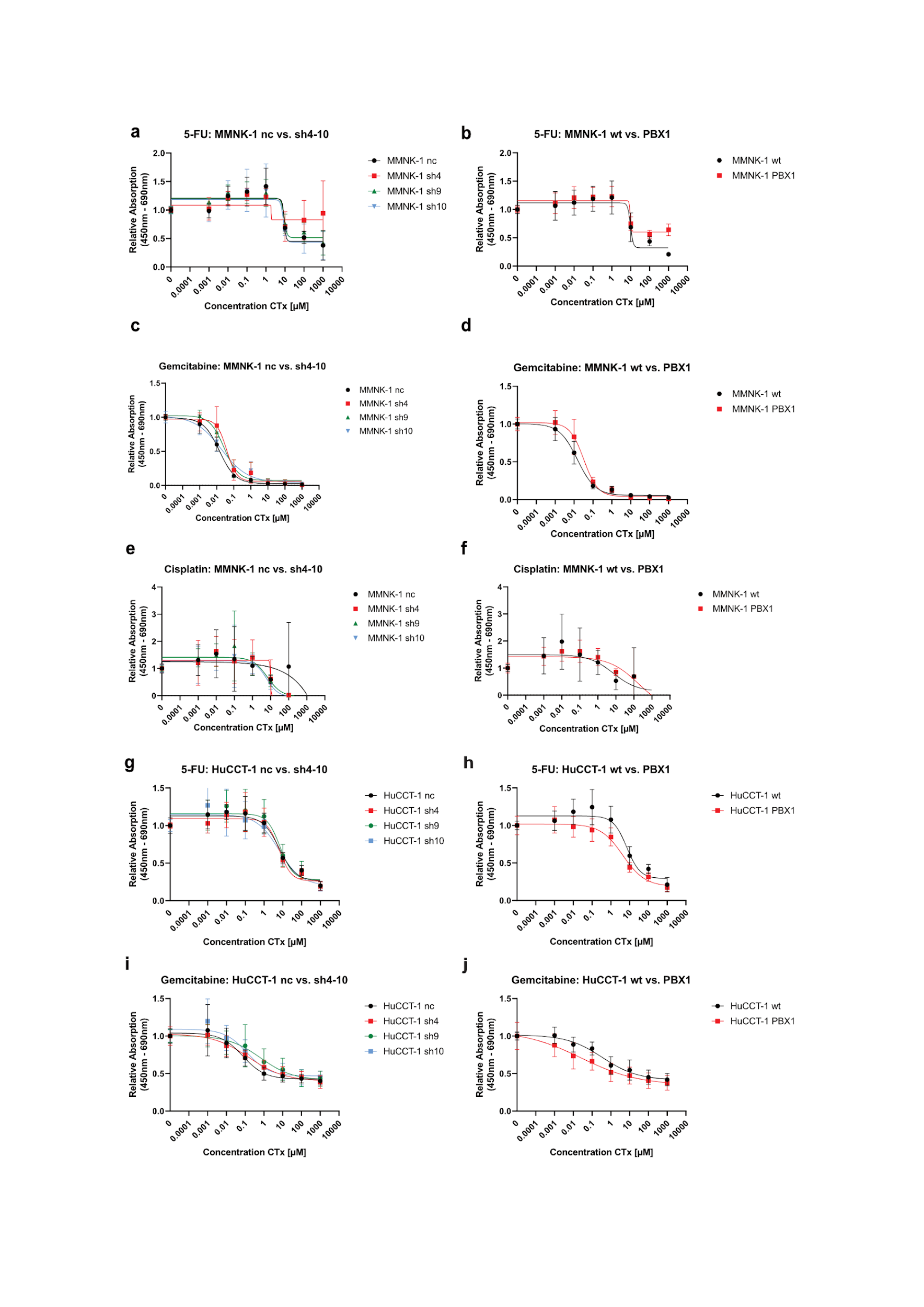


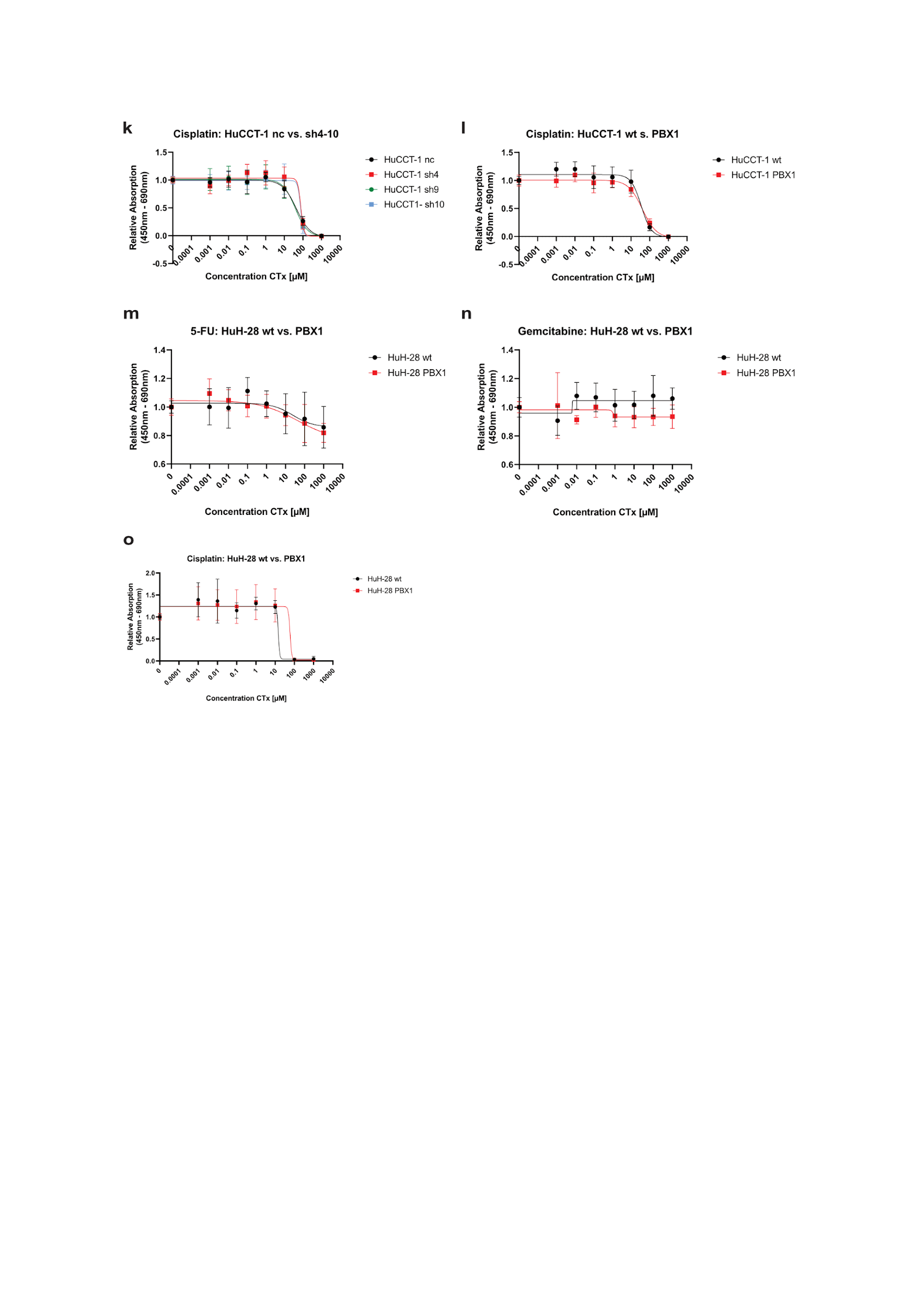


**Supplementary Figure 8.** **Chemoresistance of non-malignant and malignant bile duct cell lines depending on PBX1.** Cell lines were cultured with increasing amounts of chemotherapeutic drugs (5-FU, gemcitabine, cisplatin) that are actually used in clinical routine for the treatment of advanced iCCA. No effect was observed in either the benign MMNK-1 cells (**a-f**) other malignant HuH-28 cell line (**m-o**) when *PBX1* was either knocked-down (sh4, sh9, sh10 / control: nc (non-coding)) or upregulated (PBX1 / control: wt (wild type)). In the malignant HuCCT-1 cell line (**g-l**), *PBX1*-overexpression was associated with a reduced chemoresistance, although this phenotype was significantly different from the cells with normal *PBX1*-levels. Three independent experiments were performed in triplicate (*p<0.05).


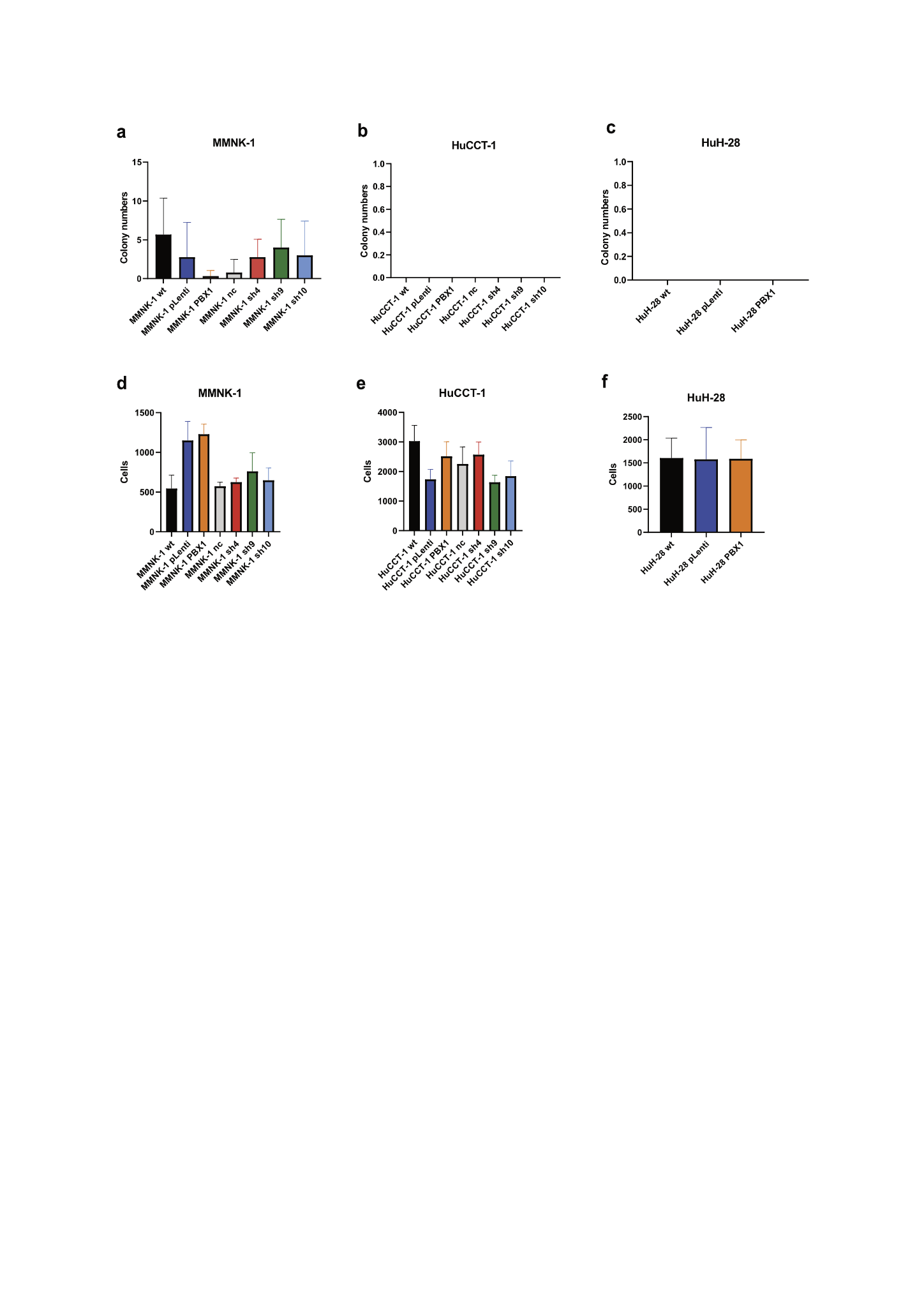


**Supplementary Figure 9.** **Colony formation assay and invasion assay of non-malignant and malignant bile duct cell lines depending on PBX1 expression.** For the colony formation assay (**a-c**), 100,000 cells per cell line were seeded into 6-well plates and cultured for 14 days before colonies (defined as more than 30 cells in a spot) were counted. Colony formation was observed only in benign MMNK-1 cells (**a**). However, no significant changes were observed. The malignant cells HuCCT-1 and HuH-28 did not show any colonies (**b-c**). In the invasion assay (**d-f**), clones of the cell lines were grown on Matrigel in tissue culture inserts with 8 µm pore size in media without fetal calf serum, separated from media containing fetal calf serum. After 24 hours, migrating cells were stained and counted. There was no significant difference in invasiveness depending on PBX1 expression in the benign MMNK-1 cells (**d**) as well as in the malignant HuCCT-1 (**e**) and HuH-28 (**f**). Three independent experiments were performed in quadruplicate (*p<0.05). Representative experiments are shown.


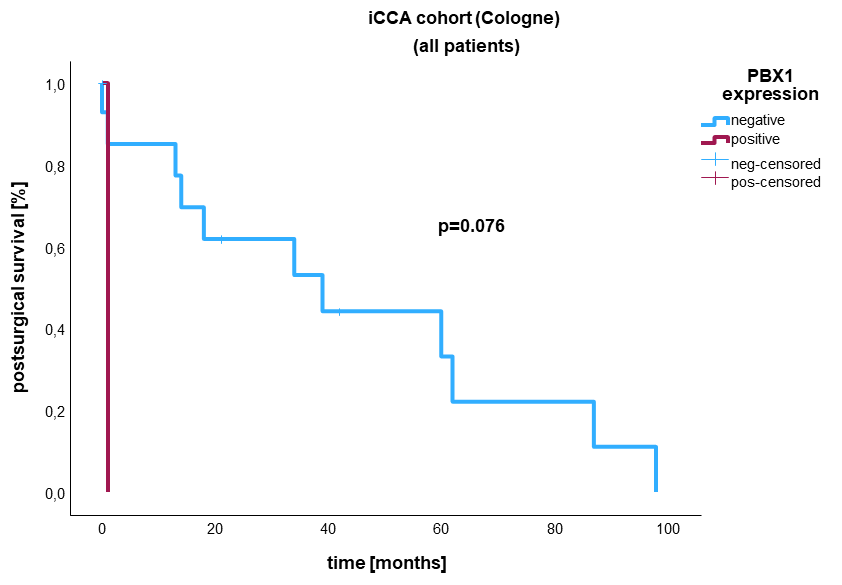


**Supplementary Figure 10.** **Prognostic impact of PBX1 in the monocentric iCCA cohort from Cologne.** Tissue microarrays (TMAs) were generated of the Cologne iCCA cohort of patients who underwent surgical resection without pervious chemotherapy or other treatment, and Kaplan-Meier analysis was performed according to PBX1 protein expression at postoperative follow-up of 15 iCCA. TMA spots were immunohistochemically stained and categorized as either samples without PBX1 expression or samples with detectable PBX1. PBX1 expression (n=1) was significantly associated with a poor postoperative prognosis compared to samples with PBX1 absence (n=14) (p=0.076).

### Supplementary Tables

**(as supplementary files)**

Supplementary Table 1 Clinical data Germany screening cohort

Supplementary Table 2 WES coverage statistics of screening cohort

Supplementary Table 3 WES mutations of screening cohort

Supplementary Table 4 WES variant validation tier 1 (Sanger seq)

Supplementary Table 5 WES variant validation tier 2 (deep NGS)

Supplementary Table 6 WES MutSigCV of screening cohort

Supplementary Table 7 SCNA screening cohort

Supplementary Table 8 SCNA gene information screening cohort

Supplementary Table 9 ASCAT ploidy screening cohort

Supplementary Table 10 GISTIC screening cohort

Supplementary Table 11 Fusion genes of screening cohort

Supplementary Table 12 Differentially expressed genes screening cohort

Supplementary Table 13 GSEA DE genes screening cohort

Supplementary Table 14 OncoImpact scores

Supplementary Table 15 Clinical data validation cohort

Supplementary Table 16 Targeted sequencing statistics validation cohort

Supplementary Table 17 Somatic SNVs targeted seq validation cohort

Supplementary Table 18 SCNAs targeted seq validation cohort

Supplementary Table 19 DE genes RNA-seq cell lines
